# HER3 promotes triple-negative breast cancer progression by upregulating PHF8 via miR-34b-5p-dependent mechanism

**DOI:** 10.1038/s41419-025-08115-9

**Published:** 2025-11-06

**Authors:** Hui Lyu, CongCong Tan, Yakun Wu, Margaret E. Larsen, Qingzhao Yu, Guobin Kang, Charles Wood, Shou-Ching Tang, Bolin Liu

**Affiliations:** 1 Department of Interdisciplinary Oncology, Stanley S. Scott Cancer Center, School of Medicine, LSU Health Sciences Center, New Orleans, LA USA; 2 Biostatistics and Data Science, School of Public Health, LSU Health Sciences Center, New Orleans, LA USA; 3 Department of Medicine, LSU LCMC Health Cancer Center, School of Medicine, LSU Health Sciences Center, New Orleans, LA USA

**Keywords:** Breast cancer, Oncogenesis

## Abstract

Triple-negative breast cancer (TNBC) is one of the most aggressive subtypes of breast cancer, with limited targeted treatment options and poor clinical outcomes. HER3 has recently emerged as a promising therapeutic target, with HER3-directed antibody–drug conjugates advancing to Phase III clinical trials for non-small cell lung cancer. However, the downstream molecular mechanisms by which HER3 promotes TNBC progression remain poorly defined. In this study, we uncovered a previously unrecognized HER3/miR-34b-5p/PHF8 signaling axis that drives TNBC cell proliferation and tumor growth. Mechanistically, HER3 activation suppresses the tumor-suppressive microRNA miR-34b-5p, resulting in the upregulation of the histone demethylase PHF8 (KDM7B), which in turn represses the expression of the CDK inhibitor p27^Kip1^ and facilitates G1–S cell cycle progression. Functional studies using shRNA-mediated knockdown and overexpression systems demonstrate that PHF8 is a critical downstream effector of HER3. PHF8 depletion phenocopied HER3 knockdown, inducing G1 arrest and suppressing colony formation and proliferation in multiple TNBC cell lines, while PHF8 overexpression rescued the inhibitory effects of HER3 loss. Furthermore, orthotopic xenograft models revealed that enforced PHF8 expression restored tumor growth suppressed by HER3 silencing in vivo. Clinically, HER3 and PHF8 expression levels were positively correlated in TNBC tissue specimens, and TCGA dataset analyses indicated that the HER3/miR-34b-5p/PHF8 axis is significantly associated with poor survival outcomes in breast cancer patients. Collectively, our findings establish a novel epigenetic regulatory circuit through which HER3 drives TNBC progression and lay the groundwork for future therapeutic strategies aimed at disrupting HER3–epigenetic crosstalk in TNBC.

## Introduction

Triple-negative breast cancer (TNBC) is a highly aggressive and heterogeneous malignancy characterized by the absence of estrogen receptor (ER)and progesterone receptor (PR), and the lack of amplification/overexpression of human epidermal growth factor receptor 2 (HER2). TNBC comprises ~10–15% of all breast cancers and is associated with poor prognosis due to its high metastatic potential and limited therapies [1–4]. Conventional treatments, including chemotherapy and radiotherapy, remain the primary therapeutic options; however, resistance to the treatments frequently occurs, contributing to the high recurrence rates and poor overall survival in TNBC patients [5–7]. Although immune checkpoint inhibitors, such as pembrolizumab and atezolizumab, have shown promising results against TNBC, especially in TNBC patients with high expression of PD-L1 or elevated tumor mutational burden [8–10], their therapeutic efficacy is hindered by challenges such as tumor heterogeneity, the complex immune microenvironment, and the development of resistance [10, 11]. In addition, PARP inhibitors, including olaparib and talazoparib, have demonstrated effectiveness in TNBC tumors with BRCA mutations by inhibiting DNA repair mechanisms [12–14]. Nevertheless, their application is limited to patients harboring specific genetic alterations, and resistance to the PARP inhibitors can develop through secondary mutations in BRCA that restore DNA repair capacity. Thus, current treatment options for TNBC remain inadequate, particularly for the patients without BRCA mutations or high expression of PD-L1. These data underscore the urgent need to elucidate the molecular mechanisms driving TNBC progression, identify new therapeutic targets, and develop effective treatments for TNBC patients.

Human epidermal growth factor receptor 3 (HER3), a member of the epidermal growth factor receptor (EGFR) family, has been associated with poor clinical outcomes and resistance to targeted therapies in various cancers, including breast cancer [15–19]. While its role in other breast cancer subtypes is well-documented [20–22], the biological function of HER3 in TNBC remains less understood. Recent studies from our lab and others have identified HER3-initiated signaling as a key driver of TNBC progression [23–25]. However, the precise mechanisms through which HER3 promotes TNBC tumor growth remain elusive. Understanding the underlying mechanisms is crucial for the development of novel therapeutic approaches against HER3-driven TNBC.

In this study, we aimed to define the molecular basis of HER3-driven TNBC progression, with a particular focus on HER3 signaling-mediated regulation of the epigenetic modifying enzyme PHF8 (PHD finger protein 8, also known as KDM7B or ZNF422), which is an important histone lysine demethylase [26–28]. Analysis of TCGA data and TNBC specimens demonstrated a positive correlation between HER3 and PHF8 expression. We investigated how HER3 signaling promoted TNBC cell proliferation in vitro and in vivo via a miRNA-dependent upregulation of PHF8. Our studies uncovered a previously unrecognized mechanism of HER3-driven TNBC growth and highlight the HER3/miR-34b-5p/PHF8 axis as a promising therapeutic target for TNBC.

## Methods

### Cell culture and reagents

Human triple-negative breast cancer (TNBC) cell lines HCC1806, MDA-MB-231, MDA-MB-468, HCC1937, and HCC70 were obtained from the American Type Culture Collection (ATCC). All cell lines were cultured in DMEM/F-12 (1:1) supplemented with 10% fetal bovine serum (FBS) and maintained in a humidified incubator at 37°C with 5% CO2. Cells were confirmed to be free of mycoplasma contamination with the MycoAlert™ Mycoplasma Detection Kit (Lonza Group Ltd., Basel, Switzerland). Primary antibodies used in western blot assays included HER3 (cat#12708, 1:1000), p-HER3 (Y1289) (cat#4791, 1:1000), p-Akt (S473) (cat#9271, 1:1000), Akt (cat#9272, 1:1000), PHF8 (cat#93801, 1:1000), p27^kip1^ (cat#3686, 1:1000), p21 (cat#2947, 1:1000), Cyclin D1 (cat# 2922, 1:1000), E2F1 (cat# 3742, 1:1000) from Cell Signaling Technology (Beverly, MA, USA), and β-actin (A3853, 1:10,000) from Sigma-Aldrich (St. Louis, MO, USA). Primary antibodies used in IHC analysis included HER3 (cat#12708, 1:100), Ki67 (cat#9027, 1:400), Cleaved-Caspase-3 (cat#9661, 1:400) from Cell Signaling Technology. PHF8 (ab84779, 1:200) from Abcam (Cambridge, United Kingdom). Heregulin (HRG-β1) was purchased from Roche (Basel, Switzerland) and used at a concentration of 25 ng/ml to activate HER3 signaling. All other reagents were obtained from Fisher Scientific (Hampton, NH, USA) unless otherwise stated.

### Lentivirus production and transduction

Lentiviruses containing shRNAs targeting HER3 were employed for specific knockdown of HER3, as previously described [24]. Two PHF8-specific shRNAs (TRCN0000118321 and TRCN0000358845) were purchased from Sigma-Aldrich and validated for specificity and efficiency. Lentiviral production and transduction of control or specific shRNAs targeting HER3 or PHF8 were conducted following standard procedures.

### RNA sequencing and gene expression analysis

Total RNA was extracted from HCC1806 cells transduced with control or HER3-specific shRNA using the RNeasy kit (Qiagen). Raw sequence data were assessed for quality using FastQC (version 0.11.9) and aggregated with MultiQC. Reads were aligned to the reference genome using HISAT2, and mapped reads were further sorted, indexed, and compressed using Samtools. Gene counts were generated with the featureCounts tool. Differentially expressed genes (DEGs) were identified using DESeq2 in R (version 4.0.4). Gene ontology (GO) enrichment analysis of DEGs was performed using the “clusterProfiler” R package. Volcano plots and bubble charts for significant pathways were generated using the “ggplot2” R package. Gene expression changes were further validated by quantitative real-time PCR (qRT-PCR).

### Cell cycle analysis

Flow cytometric analysis was performed to assess cell cycle distribution in treated and untreated cells as described previously [24]. Cells were harvested and fixed with 70% ethanol and then stained with propidium iodide (50 μg/ml) and RNase A (100 μg/ml) for 30 min at 37 °C. Cell cycle distribution was analyzed using a FACScan flow cytometer (BD Biosciences, San Jose, CA) and FlowJo software.

### Western blot assays

Western blot assays were performed to examine protein expression and activation as described previously [29]. Briefly, cells were lysed in RIPA buffer and sonicated at 4 °C. Protein concentrations in the lysates were determined using the Bradford assay. Equal amount of the protein lysates was separated by SDS-PAGE and transferred onto PVDF membranes (Bio-Rad Laboratories, Hercules, CA). Membranes were incubated with primary antibodies as specified in the figure legends.

### In vivo orthotopic tumor xenograft models

Orthotopic tumor xenograft models were established following the procedures as we described previously [24]. Six-week-old female athymic nu/nu mice (Charles River Laboratories, Wilmington, MA) were maintained in accordance with Institutional Animal Care and Use Committee (IACUC) guidelines. HCC1806-luc cells (5 × 10^5) transduced with HER3 shRNA or PHF8 cDNA expression vectors were injected into the mammary fat pads of nude mice (*n* = 5 per group). Tumor growth was monitored through tumor volume measurements and bioluminescent imaging (IVIS System). At the conclusion of the study, mice were euthanized following the approved IACUC protocol, and tumor samples were collected for immunohistochemical (IHC) analysis.

### Immunohistochemistry (IHC)

IHC assays were performed as previously described [29]. Briefly, paraffin-embedded tissue sections were subjected to antigen retrieval, blocking, incubation with primary and HRP-conjugated secondary antibodies, DAB staining, hematoxylin counterstaining, dehydration, and mounting. Images were acquired using a Nikon microscope. Staining intensity was evaluated semi-quantitatively based on both intensity and distribution: – (no staining), + (weak), ++ (moderate), and +++ (strong).

### Luciferase reporter assays

Luciferase reporter assays were performed to examine miR-34b-5p-mediated regulation of the 3’ UTR of PHF8. HEK293T cells were co-transfected with miR-34b-5p mimics or inhibitors and a construct containing either the wide type or mutant 3’ UTR of PHF8. Luciferase activity was measured 24 h after transfection according to the manufacturer’s instructions.

### Statistical analysis

All data were presented as mean ± standard deviation (SD) from at least three independent experiments. Statistical analyses were performed using the unpaired two-tailed Student’s t-test, one-way ANOVA, log-rank test, and Spearman’s rank correlation test, as appropriate, using GraphPad Prism software. A *p*-value of <0.05 was considered statistically significant.

## Results

### Specific knockdown of HER3 significantly inhibits TNBC cell proliferation by modulating cell cycle progression, potentially through the regulation of epigenetic mechanisms

**Fig. 1:**
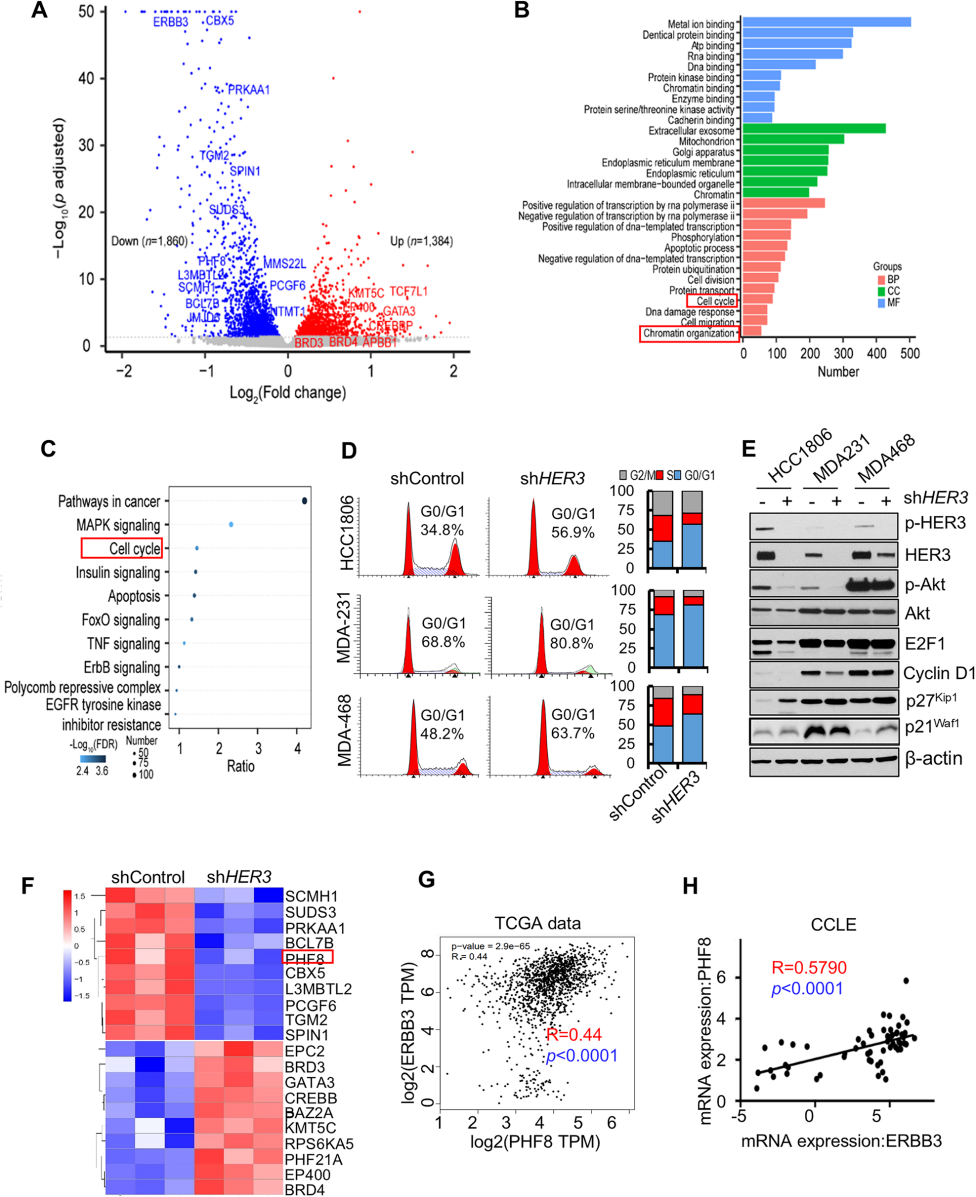
Specific knockdown of *HER3* significantly inhibits cell cycle progression and alters epigenetic regulation in TNBC cells. **A** Volcano plot for RNA-Seq analyses of differentially expressed genes (DEGs) in HCC1806 cells with or without HER3 depletion. Each point represented the average value of one transcript in three replicates. The difference was considered significant for adjusted *p* < 0.05 (red for upregulated genes, blue for downregulated genes, and gray for non-significant genes). The indicated genes were involved in chromatin organization and histone modification. **B** Gene Ontology (GO) enrichment analysis of the DEGs. Top GO terms were represented by gene count >50, with FDR < 0.05 in Molecular Function (MF), Cellular Component (CC) and Biological Process (BP). **C** KEGG pathway analysis of the DEGs. **D**, **E** HCC1806, MDA-MB-231 (MDA-231), and MDA-MB-468 (MDA-468) cells were infected with lentivirus containing either control shRNA (shControl) or specific shRNA against HER3 (sh*HER3*) for 48 h. Cell cycle distribution was analyzed with flow cytometry assays (**D**). Western blot analyses were performed to examine p-HER3, HER3, p-Akt, Akt, E2F1, Cyclin D1, p27^kip1^, p21^waf1^, and β-actin (**E**). **F** The heat map showed the top ten (down- or up-regulated) genes involved in chromatin organization and histone modification. Analysis of TCGA (**G**) and CCLE (**H**) datasets revealed a significantly positive correlation between *HER3* and *PHF8* expression in breast cancer specimens and cell lines, respectively.

To identify crucial downstream mediators of HER3 signaling in TNBC, we silenced HER3 expression by a specific shRNA in HCC1806 cells and performed RNA-sequencing (RNA-Seq) analysis of HCC1806-conshRNA vs HCC1806-HER3shRNA cells. Compared to the control shRNA group, specific knockdown of HER3 led to significant upregulation of 1384 genes and downregulation of 1860 genes (Fig. 1A). Gene Ontology (GO) enrichment analysis of the differentially expressed genes (DEGs) revealed key biological processes, including cell cycle progression and chromatin remodeling, that were significantly altered upon HER3 knockdown (Fig. 1B). KEGG pathway analysis also identified several pathways related to cell cycle, apoptosis, and gene transcriptional regulation that were affected by HER3 knockdown (Fig. 1C). We therefore hypothesized that regulation of cell cycle progression and gene transcription associated with chromatin remodeling might be critical for HER3 promotion of TNBC cell proliferation. To test this hypothesis, we utilized specific shRNA to downregulate HER3 expression in three TNBC cell lines (HCC1806, MDA-MB-231 and MDA-MB-468). Colony formation assays showed a significant reduction in colony formation ability upon HER3 knockdown, indicating a substantial impairment in proliferative capacity (Supplementary Fig. S1A). Specific knockdown of HER3 potently induced cell cycle G1 arrest in all three cell lines tested (Fig. 1D). This was accompanied by reduced expression of Cyclin D1 and E2F1, and a marked increase in p27^kip1^, a critical CDK inhibitor in G1-S transition. Additionally, depletion of HER3 markedly reduced the levels of phosphorylated Akt (p-Akt), a key downstream molecule of HER3 signaling (Fig. 1E). This data suggests that HER3 plays a critical role in regulating cell cycle progression of TNBC cells. Next, we examined whether HER3 played a role in epigenetic regulation via chromatin remodeling. Further analysis of our RNA-Seq data discovered that specific knockdown of HER3 dramatically inhibited expression of several epigenetic modifiers, with PHF8 as one of the most downregulated ones (Fig. 1F). PHF8 has been shown to be highly expressed in BC tumors compared to normal breast tissues and other tumor types (Supplementary Fig. S1B, C). It is a key histone lysine demethylase that plays a pivotal role in epigenetic regulation by modifying chromatin structure to alter gene transcription. PHF8 has been implicated in various types of human cancers, including BC, where it contributes to tumor progression by regulating cell cycle progression, DNA damage response, and oncogenic expression. Moreover, bioinformatics analysis of TCGA (Fig. 1G) and CCLE (Fig. 1H) datasets showed a significantly positive correlation between HER3 and PHF8 expressions in BC clinical samples and cell lines, respectively. Thus, our studies support that PHF8 may serve as a key downstream mediator of HER3 signaling in the regulation of cell cycle progression of TNBC cells.

### PHF8 plays a critical role in HER3-driven TNBC cell cycle progression via modulation of p27^kip1^

**Fig. 2:**
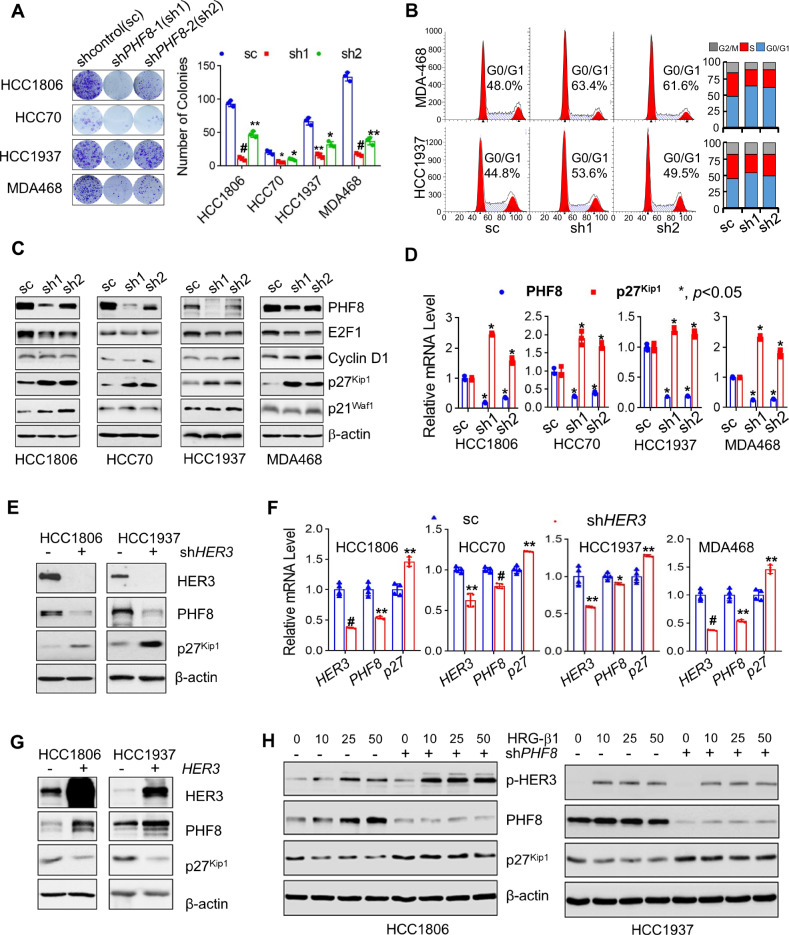
Specific knockdown of PHF8 not only exhibits similar effects as HER3 depletion to induce cell cycle G1 arrest and upregulatep27^kip1^, but also abrogates activation of HER3-mediated downregulation of p27^kip1^ in TNBC cells. TNBC cell lines (HCC1806, HCC70, HCC1937, and MDA-MB-468) were transiently infected with lentivirus containing either control shRNA (sc) or specific shRNAs targeting PHF8 (sh1 and sh2). **A** Cells were seeded onto 6-well plates for colony formation assays. Representative images of the cell colonies were captured using a digital camera after crystal violet staining (left panel), and the colony numbers were quantified using ImageJ software (right panel). Error bars represent the standard deviation (SD). *, *p* < 0.05; **, *p* < 0.01; #, *p* < 0.005. **B** Cell cycle progression was examined by flow cytometry analysis. **C** Western blot was assays were conducted to detect PHF8, E2F1, Cyclin D1, p27^kip1^, p21^waf1^, and β-actin. **D**, RT-qPCR was performed to measure *PHF8* and *p27*^*kip1*^ mRNA expression. **E**, **F** HCC1806 and HCC1937 cells were infected with lentivirus containing either control shRNA (-) or *HER3*-specific shRNA (+). After 48 h, cells were collected and subjected to western blot analysis of HER3, PHF8, p27^kip1^, and β-actin (**E**). RT-qPCR analysis was performed to quantify the mRNA expression levels of *HER3, PHF8, and p27*^*kip1*^ (**F**). **G** HCC1806 and HCC1937 cells with ectopic expression of HER3 were subjected to western blot analysis of HER3, PHF8, p27^kip1^, and β-actin. **H** TNBC cells infected with lentivirus containing either control shRNA (-) or *PHF8*-specific shRNA (+) were stimulated with HRG-β1 (25 ng/ml) for 24 h. Cells were collected and examined by western blot analysis of p-HER3, PHF8, p27^kip1^, and β-actin.

Our identification of PHF8 as a potential key downstream mediator of HER3 signaling in TNBC suggests that PHF8 expression critically contributes to HER3-driven TNBC cell proliferation and cell cycle progression. Thus, we first assessed the impact of silencing PHF8 on cell proliferation and found that specific knockdown of PHF8 significantly impaired cell proliferative capacity, as evidenced by a marked reduction of colony numbers in all TNBC cell lines tested (Fig. 2A).

Next, we examined the role of PHF8 in the regulation of cell cycle progression. MDA-MB-468 and HCC1937 cells were infected with lentivirus containing either scramble control shRNA or PHF8-targeting shRNA. Like the effects observed with downregulation of HER3 (Fig. 1D), specific knockdown of PHF8 led to a significant accumulation of cells in the G1 phase (Fig. 2B), indicating that PHF8 depletion potently inhibited TNBC cell cycle progression. PHF8 depletion increased the expression of p27^kip1^ but decreased the expression levels of both Cyclin D1 and E2F1 (Fig. 2C). RT-qPCR confirmed a significant reduction in PHF8 mRNA levels upon shRNA transduction. Interestingly, we also found that specific knockdown of PHF8 resulted in a dramatic increase in the mRNA expression of p27^kip1^ (Fig. 2D). These findings suggest that PHF8 plays a role in promoting TNBC cell cycle progression likely through regulation of p27^kip1^ expression.

To determine whether PHF8 plays a critical role in HER3-driven TNBC cell cycle progression, HER3 was transiently knocked down in HCC1806 and HCC1937 cells. HER3 silencing markedly reduced PHF8 protein levels and increased p27^Kip1^ expression (Fig. 2E). RT-qPCR confirmed that HER3 knockdown significantly decreased PHF8 mRNA and upregulated p27^Kip1^ transcripts across all TNBC cell lines tested (Fig. 2F). Conversely, overexpression of HER3 in HCC1806 and HCC1937 cells upregulated PHF8 expression and downregulated p27^kip1^ levels (Fig. 2G). Moreover, stimulation of TNBC cells with HRG-β1, which is a ligand for HER3 and can activate HER3 signaling, increased PHF8 expression but decreased the protein levels of p27^kip1^ in a dose-dependent manner (Fig. 2H and Supplementary Fig. S2). However, PHF8 depletion effectively blocked the effects of HRG-β1 on PHF8 and p27^kip1^ (Fig. 2H). Collectively, our data strongly support PHF8 critically contributes to HER3-driven TNBC cell proliferation and cell cycle progression via modulation of p27^kip1^.

### The expression of PHF8 is required for HER3-driven TNBC cell proliferation and cell cycle progression

**Fig. 3:**
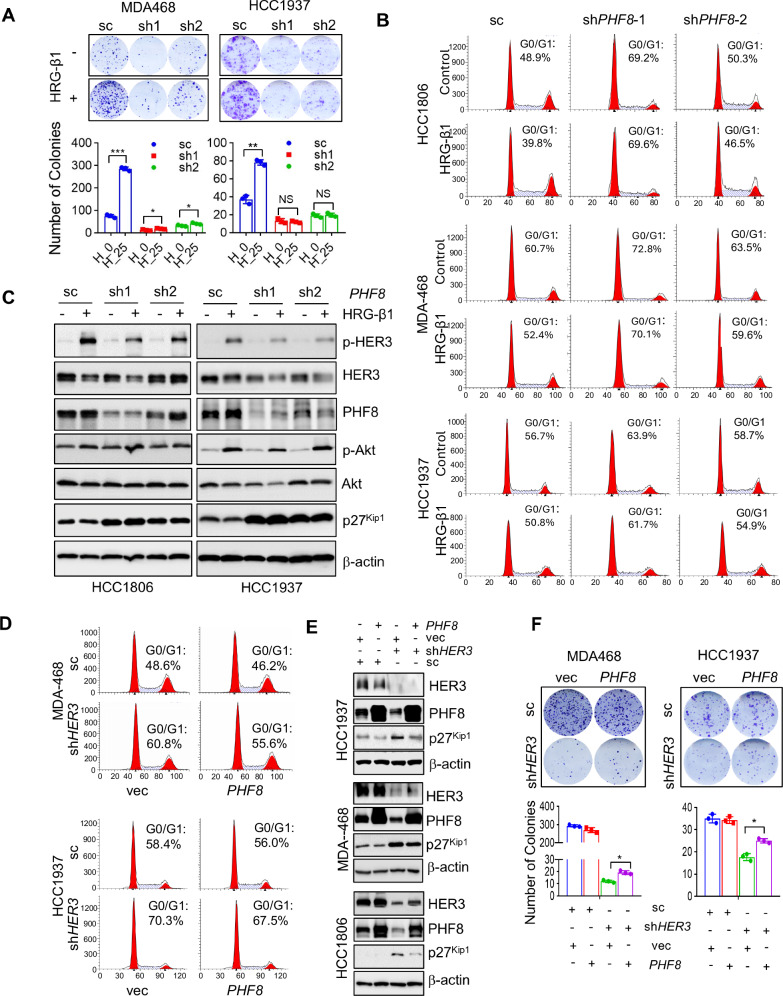
PHF8 is required for activation of HER3 signaling-mediated promotion of colony formation, cell cycle progression, and downregulation of p27^kip1^, whereas ectopic expression of PHF8 partially reverses HER3 depletion-induced cell cycle G1 arrest, upregulation of p27^kip1^, and inhibition of colony formation in TNBC cells. **A** MDA-MB-468 and HCC1937 cells infected with lentivirus containing either control shRNA (sc) or *PHF8*-specific shRNA (sh1 and sh2) was seeded into 6-well plates for colony formation assays. Cells were stimulated with or without HRG-β1 (25 ng/ml). Representative images of cell colonies were captured, and the colony numbers were quantified using ImageJ software. Error bars represent the standard deviation (SD) from three independent experiments. *, *p* < 0.05; **, *p* < 0.01; ***, p < 0.001. **B**, **C** TNBC cells were seeded into 6-well plates and transduced with lentivirus containing either control shRNA (sc) or *PHF8*-specific shRNA (sh1 and sh2) for 24 h. Subsequently, the cells were stimulated with or without HRG-β1 (25 ng/ml) for additional 24 h. Cells were harvested for flow cytometry analysis of cell cycle distribution (**B**). Cells were collected for western blot analysis of p-HER3, HER3, PHF8, p-Akt, Akt, p27^kip1^, and β-actin (**C**). **D**–**F** TNBC cells were infected with lentivirus containing either control shRNA (sc) or *HER3*-specific shRNA (sh*HER3*) to achieve knockdown of HER3. The cells were then transfected with either a control vector (vec) or *PHF8* cDNA expression vector (*PHF8*). The resulting cells were analyzed by flow cytometry to assess cell cycle distribution (**D**), by western blot assays to examine HER3, PHF8, p27^kip1^, and β-actin (**E**), and by colony formation assays. Representative images of the colonies were captured, and the colony numbers were quantified using ImageJ software (**F**). Error bars represent the standard deviation (SD) from three independent experiments. Statistically significant differences were assessed, with *, *p* < 0.05 compared to the control.

To determine whether PHF8 played an essential role in HER3-induced cell proliferation, we conducted colony formation assays in TNBC cells following PHF8 depletion. TNBC cells transduced with PHF8-targeting shRNA (sh1/sh2) or control shRNA (sc) were seeded into six-well plates and cultured in the presence or absence of HRG-β1. As shown in Fig. 3A, specific knockdown of PHF8 abolished the stimulative effects of HRG-β1 on colony formation in both MDA-MB-468 and HCC1937 cells, suggesting that the expression of PHF8 was required for HER3 signaling-mediated promotion of TNBC cell proliferation. In addition, HRG-β1-induced TNBC cell growth was significantly reduced upon silencing of PHF8 (Supplementary Fig. S3A). Moreover, specific knockdown of PHF8 completely inhibited HRG-β1-promotted cell cycle G1-S transition in all TNBC cells tested (Fig. 3B), supporting that PHF8 played an essential role in HER3-driven TNBC cell cycle progression. Stimulation of TNBC cells with HRG-β1 increased PHF8 expression but led to an obvious reduction in the expression of p27^kip1^. However, PHF8 depletion markedly attenuated the effects of HRG-β1 on p27^kip1^, rather resulting in upregulation of p27^kip1^ (Fig. 3C and Supplementary Fig. S3B). Activation of HER3 signaling by HRG-β1 was evidenced by the increased levels of p-HER3 and p-Akt. On the contrary, ectopic expression of PHF8 partially rescues the inhibitory effects of HER3 depletion on cell cycle progression and colony formation. Flow cytometry assays showed that specific knockdown of HER3 potently induced cell cycle G1 arrest, whereas overexpression of PHF8 seemed to partially rescue the G1 arrest-caused by silencing of HER3 in MDA-MB-468 and HCC1937 cells, but not in HCC1806 cells (Fig. 3D and Supplementary Fig. S4A). Ectopic expression of PHF8 effectively counteracted HER3 depletion-induced upregulation of p27^kip1^ protein expression in the TNBC cells (Fig. 3E). We also found that overexpression of PHF8 partially reversed the reduction of colony formation-caused by HER3 depletion (Fig. 3F and Supplementary Fig. S4B). These results collectively indicate that the expression of PHF8 is required for HER3-driven TNBC cell cycle progression and proliferation.

### Ectopic expression of PHF8 abrogates the inhibitory effects of HER3 depletion on TNBC tumor growth in vivo

**Fig. 4:**
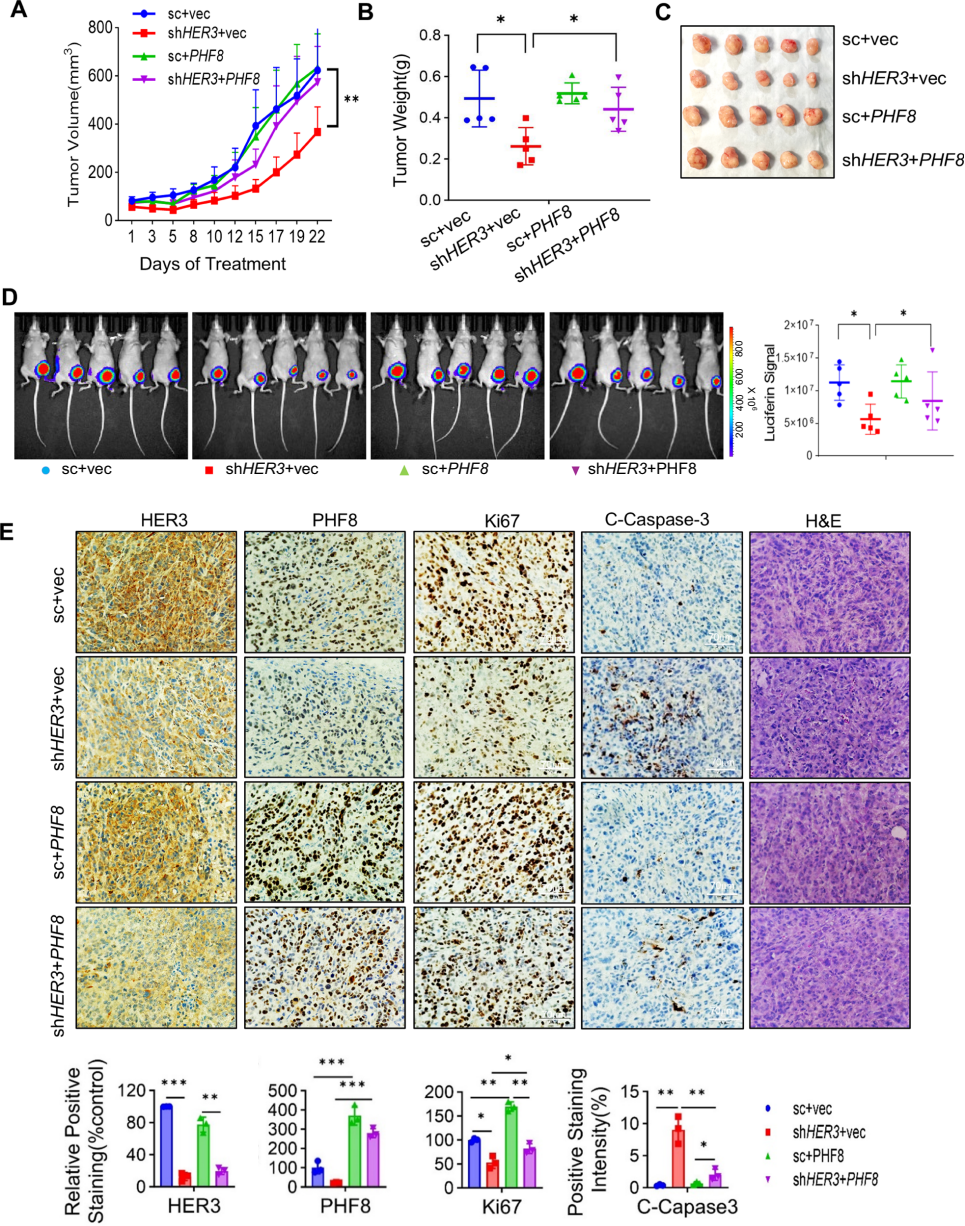
Ectopic expression of PHF8 significantly attenuates the inhibitory effects of HER3 depletion on TNBC tumor growth in orthotopic tumor models. HCC1806-luc cells (5 × 10^5^) were transiently transduced with either control shRNA (sc) or *HER3*-specific shRNA (sh*HER3*). The cells were then transfected with either a control vector (vec) or *PHF8* cDNA expression vector (*PHF8*). The resulting cells were orthotopically inoculated into the mammary fat pads of nude mice to establish tumor xenografts (*n* = 5). **A** Tumor growth curves were plotted using average tumor volumes within each group at the indicated time points. A two-tailed student’s t-test was used for statistical analysis. Bars, SEM. **, *p* < 0.01. **B**, **C** The mammary tumors were dissected, imaged as indicated and measured for weight. Bars, SD. *, *p* < 0.05. **D** Bioluminescent imaging of the mammary tumors was performed with IVIS Spectrum system. The luciferase signal intensity from the tumors was quantified and plotted. Error bars represent the standard deviation (SD)*, *p* < 0.05. **E** Formalin-fixed paraffin-embedded sections of the tumors were analyzed by H&E staining and IHC for HER3, PHF8, Ki67, and Cleaved Caspase-3 (C-Caspase-3). Scale bar, 70 µm. Quantification of IHC assays was performed using ImageJ, with statistical analysis conducted using two-way ANOVA (*, *p* < 0.05; **, *p* < 0.01; ***, *p* < 0.001). Error bars represent SD.

To evaluate the functional significance of PHF8 in HER3-driven TNBC tumor growth in vivo, we conducted experiments with orthotopic tumor xenograft models. HCC1806 cells labelled with luciferase (HCC1806-Luc) were transduced with lentivirus carrying either control shRNA (sc) or HER3-targeting shRNA (sh*HER3*), along with an empty vector (vec) or a PHF8 cDNA expression vector (*PHF8*). The resulting cells were divided into four experimental groups: sc+vec, sh*HER3*+vec, sc+*PHF8*, and sh*HER3* + *PHF8*. The cells of each group were orthotopically injected into the mammary fat pads of nude mice (*n* = 5). Tumor growth was monitored over time. We discovered that specific knockdown of HER3 significantly suppressed TNBC tumor growth. However, ectopic expression of PHF8 at least partially rescued tumor growth-inhibited by HER3 depletion (Fig. 4A). These data indicate that PHF8 plays a critical role in promoting HER3-driven TNBC tumor growth in vivo. Consistently, measurements of tumor weight and bioluminescence imaging of the tumors confirmed that overexpression of PHF8 restored tumor growth in HER3-depleted TNBC cells (Fig. 4B–D). Moreover, IHC analysis of tumor sections revealed a marked reduction of PHF8 expression in HER3-depleted tumors, accompanied by a significant decrease of Ki67 and increase of cleaved caspase-3, indicative of reduced proliferation and enhanced apoptosis, respectively. Importantly, overexpression of PHF8 significantly reversed the effects of HER3 depletion on cleaved caspase-3 and Ki67 expression, suggesting that elevated expression of PHF8 effectively rescued TNBC cell proliferation and blocked apoptosis-induced by HER3 depletion (Fig. 4E). These findings highlight PHF8 as a key downstream effector of HER3 signaling in promoting TNBC tumor growth in vivo.

### HER3 signaling upregulates PHF8 via suppression of miR-34b-5p in TNBC cells

**Fig. 5:**
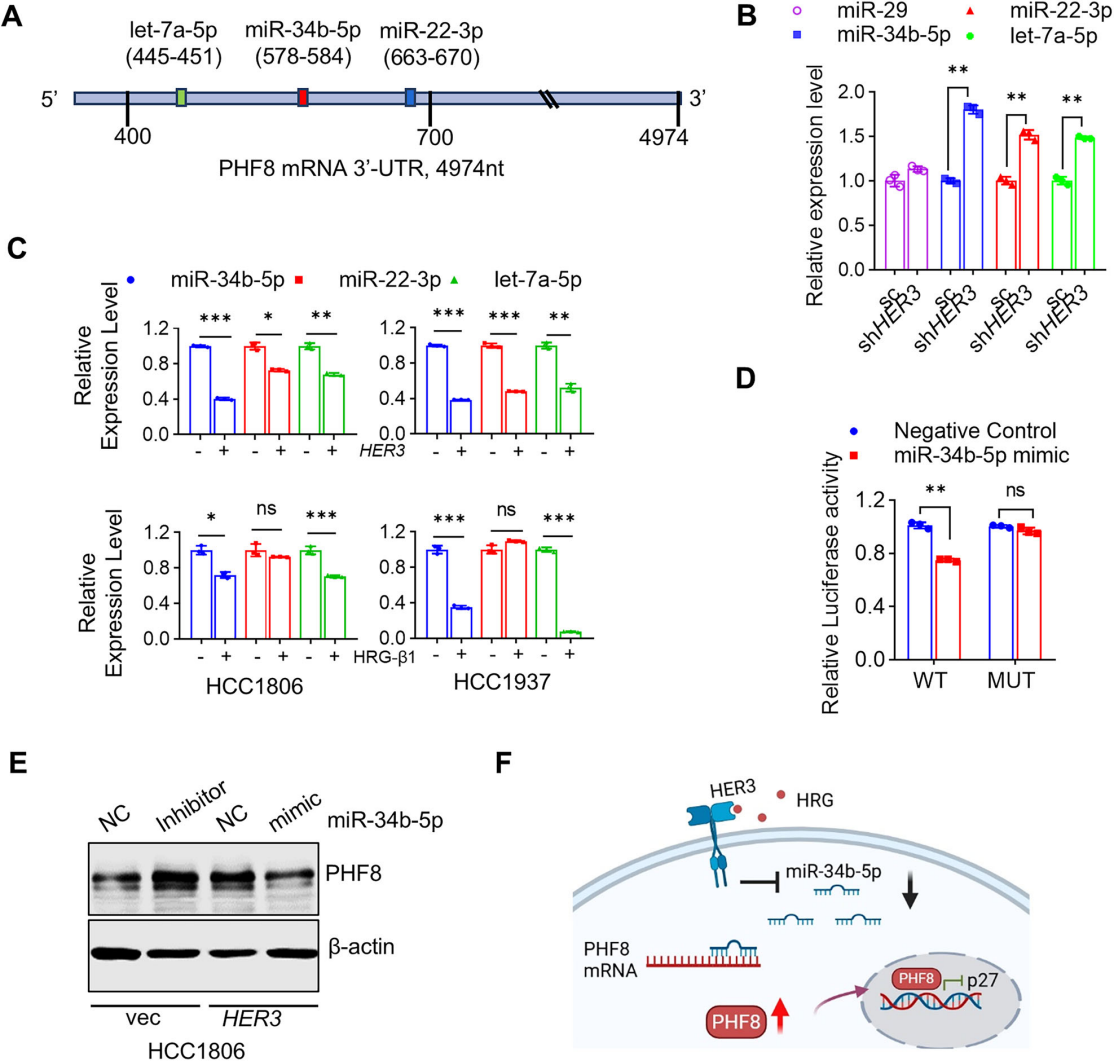
HER3 signaling upregulates PHF8 via suppression of miR-34b-5p in TNBC cells. **A** Schematic representation of the predicted miRNAs binding sites within 3’-UTR of *PHF8* mRNA. **B** HCC1806 cells with specific knockdown of HER3 were analyzed by RT-qPCR to measure the expression levels of indicated miRNAs. **C** HCC1806 and HCC1937 cells with ectopic expression of HER3 or stimulated with HRG-β1(25 ng/ml) for 24 h. RT-qPCR was performed to measure miRNA expressions levels. **D** luciferase activity was detected using HEK 293 T cells, which were co-transfected with a miRNA mimic control(Negative Control) or miR-34b-5p mimic along with a luciferase reporter construct containing either the wild-type (WT) or mutated (MUT) miR-34b-5p binding site of the 3’-UTR of *PHF8* mRNA. **E** HCC1806-pLEX (vec) cells were transfected with either miRNA inhibitor control (NC) or specific inhibitor for miR-34b-5p. The HER3-overexpressing HCC1806 cells (HER3) were transfected with either a miRNA mimic control (NC) or miR-34b-5p mimic. The resulting cells were analyzed by western blot assays to examine PHF8 and β-actin. **F** Proposed model of the mechanism by which HER3 signaling upregulates PHF8 via suppression of miR-34b-5p to promote proliferation and inhibit p27^kip1^ expression in TNBC cells.

HER3-initiated signaling is known to activate multiple oncogenic pathways, including those involved in epigenetic modifications and/or post-transcriptional regulation of gene expression [30, 31]. Given the emerging role of microRNAs (miRNAs) as key modulators of oncogenic signaling [32–36], we hypothesized that HER3 signaling may upregulate PHF8 in TNBC via a miRNA-dependent mechanism. TargetScan analysis identified several miRNAs, including Let-7a-5p, miR-34b-5p, and miR-22-3p as potential regulators of PHF8 expression since they all have predicted binding sites on the 3’UTR of PHF8 mRNA (Fig. 5A). Specific knockdown of HER3 in HCC1806 cells resulted in a significant increase in the expression of miR-34b-5p, miR-22-3p, and let-7a-5p, whereas the expression levels of miR-29, a negative control miRNA, which was not predicted to bind to the 3’UTR of PHF8 mRNA, remained unchanged (Fig. 5B). Conversely, overexpression of HER3 or HRG-β1 stimulation markedly decreased the expression levels of miR-34b-5p and let-7a-5p in both HCC1806 and HCC1937 cells (Fig. 5C). During the subsequent examinations with additional TNBC cell lines, we observed miR-34b-5p as the most consistently regulated miRNA following HER3 activation or depletion (data not shown).

In addition, numerous studies have established miR-34b-5p as a crucial tumor-suppressive miRNA regulating proliferation, apoptosis, and metastasis in various human cancers [37–39]. Hence, we selected miR-34b-5p for further investigation. To determine if miR-34b-5p could directly bind to the 3’UTR of PHF8 mRNA, we constructed luciferase reporter vectors-driven by PHF8 3’UTR containing either miR-34b-5p wild-type or mutant binding site. Our results showed that miR-34b-5p mimics significantly reduced the luciferase activity-induced by the wild-type, but not mutant PHF8 3′ UTR(Fig. 5D), suggesting that miR-34b-5p downregulates PHF8 in TNBC cells likely via targeting its 3′ UTR. Furthermore, repression of miR-34b-5p with a specific inhibitor elevated the protein levels of PHF8 in HCC1806 cells, mimicking the effect of ectopic expression of HER3. In contrast, increased expression of miR-34b-5p via mimic transfection completely reversed HER3 overexpression-mediated upregulation of PHF8 (Fig. 5E). Taken together, these findings demonstrate that HER3 signaling enhances PHF8 expression via suppression of miR-34b-5p in TNBC cells. A proposed mechanistic model (Fig. 5F) illustrates that activation of HER3 signaling results in upregulation of PHF8 via suppression of miR-34b-5p, which in turn inhibits p27^kip1^ expression, ultimately promoting TNBC cell proliferation and cell cycle progression.

To further investigate the clinical relevance of the HER3/miR-34b-5p/PHF8 axis, we analyzed the expression correlations between miR-34b-5p and PHF8 or HER3 using the TCGA pan-cancer dataset. A significant negative correlation between miR-34b-5p and PHF8 was observed in several cancer types, suggesting that PHF8 may be a potential target of miR-34b-5p (Supplementary Fig. S5A). However, this correlation was not observed in the overall breast cancer cohort. We plan to conduct future studies to assess the miR-34b-5p expression profile in our TNBC specimens and evaluate its correlation with PHF8. Analysis of miR-34b-5p and HER3 across multiple cancer types revealed predominantly negative correlations in several malignancies, with weaker positive associations in a subset of tumor types, indicating cancer-specific regulatory patterns (Supplementary Fig. S5B). In breast cancer, we observed a very weak positive correlation between miR-34b-5p and HER3 expression (R = 0.062, p = 0.04). As noted previously, future investigations should prioritize subtype-stratified analyses, with particular focus on TNBC.

### Positive correlation of HER3 and PHF8 is observed in TNBC specimens and the HER3/miR-34b-5p/PHF8 axis is clinically relevant in the survival outcomes of breast cancer patients

**Fig. 6:**
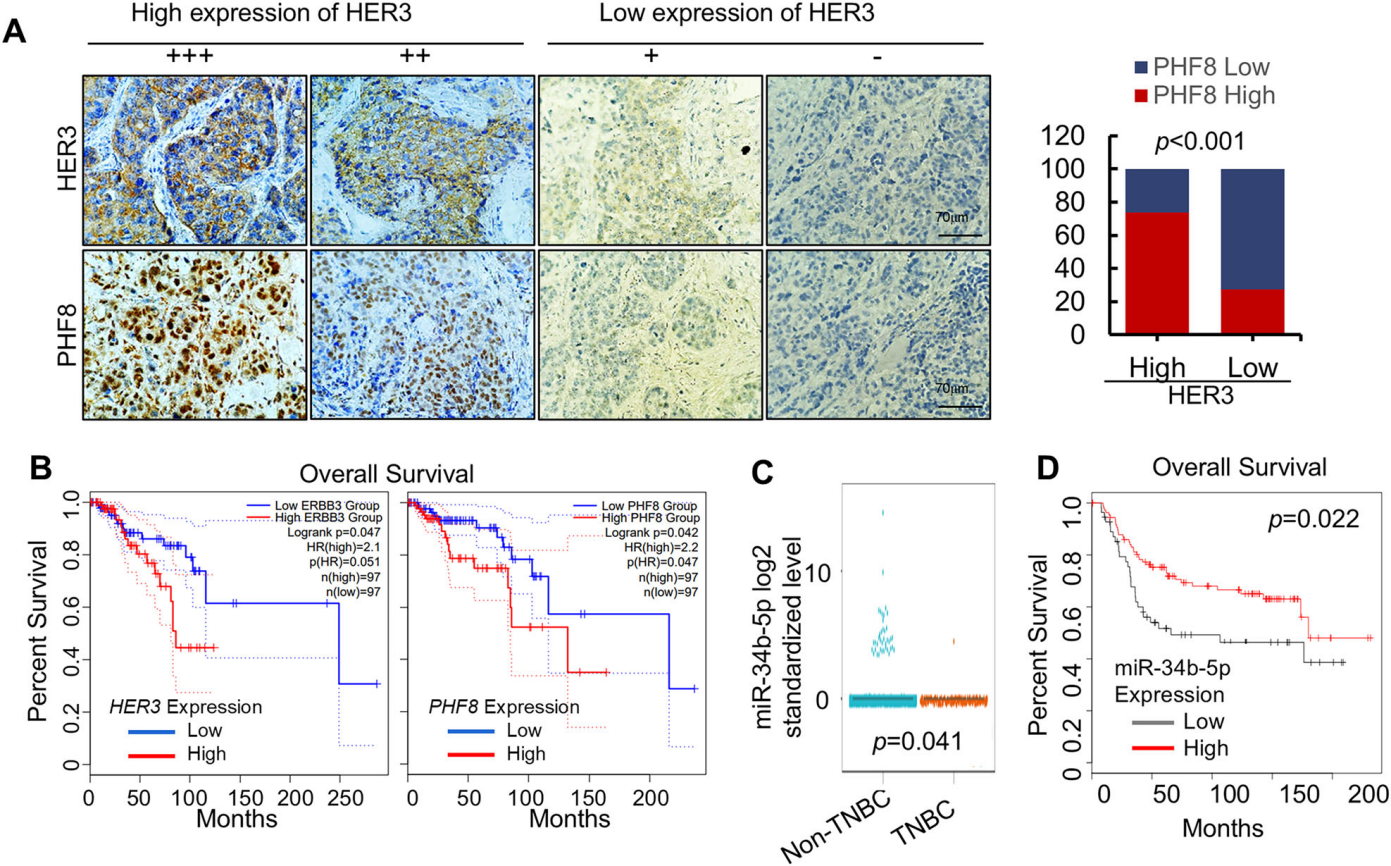
HER3 and PHF8 expressions are positively correlated in TNBC specimens and the HER3/miR-34b-5p/PHF8 signaling axis significantly associates with survival outcomes in breast cancer patients. **A** Representative images of IHC staining for HER3 and PHF8 in TNBC specimens. A significantly positive correlation between HER3 and PHF8 was observed in TNBC specimens. **B** Kaplan-Meier survival curves showed that breast cancer patients with tumors expressing high mRNA levels of *HER3* or *PHF8* exhibited much worse overall survival than those patients with tumors expressing low mRNA levels of *HER3* or *PHF8*, respectively. **C** The expression level of miR-34b-5p was significantly lower in TNBC tumors (orange, *n* = 254) than that in non-TNBC tumors (blue, *n* = 3777). Data was acquired from Breast Cancer Gene-Expression Miner v5.2 website. **D** Kaplan-Meier survival curves of overall survival of breast cancer patients from TCGA datasets. The log-rank tests were used to compare overall survival of breast cancer patients with tumors expressing high versus low expression levels of miR-34b-5p.

To investigate the clinical relevance of our findings, we examined the expressions of HER3 and PHF8 in a cohort of 91 TNBC specimens. Immunohistochemical analysis of tumor samples revealed that 35.2% (32/91) of them exhibited high HER3 expression, while 45.1% (41/91) of them showed high PHF8 expression. Pearson’s Chi-squared correction analysis demonstrated a significant positive correlation between HER3 and PHF8 expression (*p* = 6.134e-05), further supporting their functional interaction in TNBC progression. Representative images illustrating varying expression levels of HER3 and PHF8 were shown in Fig. 6A and Supplementary Fig. S6. Additionally, Kaplan-Meier survival analysis of TCGA datasets indicated that elevated mRNA expression of both HER3 and PHF8 was significantly associated with poor overall survival in breast cancer patients (Fig. 6B). Moreover, the expression of miR-34b-5p was much lower in TNBC samples than that in non-TNBC samples (Fig. 6C) and the reduced miR-34b-5p levels were significantly associated with poor overall survival in breast cancer patients (Fig. 6D). Our data strongly supports that the HER3/miR-34b-5p/PHF8 axis potentially serves as relevant biomarkers predictive for the survival outcomes of breast cancer patients.

## Discussion

HER3 emerges as a compelling therapeutic target in various human cancers, including TNBC, due to its role in activating multiple oncogenic signaling pathways [21, 24, 40, 41]. Unlike other HER family members, HER3 lacks intrinsic kinase activity and exerts its oncogenic effects through heterodimerization with other receptor tyrosine kinases, most notably EGFR and HER2, leading to potent activation of the PI3K/Akt pathway [42, 43]. Despite its relatively lower expression in TNBC compared to HER2-psitive and luminal breast cancers, accumulating evidence suggests that HER3 remains functionally relevant in TNBC tumorigenesis, especially in the presence of its ligand, HRG-β1 [22, 23, 44–46]. Interestingly, our analysis of TCGA datasets reveals that HRG-β1 is highly expressed in TNBC compared to other breast cancer subtypes (Supplementary Fig. S7), consistent with recent studies highlighting the important role of HRG-β1 in TNBC [47, 48]. These findings strongly support a critical role for HER3 signaling in the promotion of TNBC progression. Notably, we previously showed that targeting HER3 with our newly developed monoclonal antibody 4A7 significantly enhanced the efficacy of chemotherapy in the preclinical models of TNBC, suggesting the therapeutic potential of HER3 inhibition in this aggressive subtype of breast cancer [24, 25]. Furthermore, recent advancements in antibody-drug conjugates (ADCs) have reinforced the significance of HER3 as a viable therapeutic target. Several HER3-directed ADCs, such as patritumab deruxtecan (HER3-DXd), have shown promising preclinical and clinical activity in a variety of human malignancies, including TNBC [49, 50]. These ADCs leverage HER3’s tumor-specific expression to deliver cytotoxic agents directly to cancer cells, thereby minimizing systemic toxicity. Given these advancements, identification of novel HER3-targeted strategies, either as monotherapies or in combination with existing treatment modalities, warrants further investigation in TNBC [51].

Beyond directly targeting HER3, blocking its key downstream signaling or mediators represents an alternative complementary strategy. In the current study, we identified PHF8 as a novel and significant mediator of HER3 signaling in TNBC. Interestingly, an early study demonstrated a synergy between PHF8 and HER2 signaling in the development HER2-positive breast cancer, revealing significant therapeutic implications for targeting PHF8 in HER2-positive breast cancer to overcome therapy resistance [52]. Given the well-established role of HER3 in HER2-positive breast cancer progression and therapeutic resistance, it is conceivable to hypothesize that PHF8 potentially acts as a key downstream mediator of HER3 signaling as well. Indeed, we observed that specific knockdown of HER3 markedly decreased PHF8 expression in TNBC and non-small cell lung cancer cells (data not shown). As a critical histone lysine demethylase, PHF8 has been implicated in multiple oncogenic processes, including cell cycle regulation, transcriptional activation, and chromatin remodeling [53–58]. It contains a plant homeodomain (PHD) finger domain, which is a common motif for recognizing methylated lysine residues on histones and other proteins. PHF8 also contains a Jumonji C (JmjC) domain, which is known for its demethylase activity. These structural features enable PHF8 to interact with histones and other chromatin-associated factors to regulate gene transcription [58–60]. PHF8 has been widely recognized as a transcriptional activator due to its histone demethylase activity, which primarily targets repressive histone marks such as H3K9me1/me2 and H4K20me1. By removing these epigenetic modifications, PHF8 facilitates an open chromatin state, allowing transcription factors and RNA polymerase II to access promoter regions and drive gene expression [27, 61–63]. While PHF8 is well characterized as a transcriptional activator, our current studies suggest that PHF8 may also contribute to transcriptional repression, thereby promoting cancer progression. Our data is supported by a recent report showing that PHF8 interacts with the transcription factor YY1, functioning as a co-repressor to inhibit a significant subset of nuclear-encoded electron transport chain (ETC) genes. This repression in turn drives mitochondrial reactive oxygen species (mROS) production, contributing to cancer development [64]. We discovered that PHF8 functioned as a transcriptional repressor to suppress p27^kip1^ expression in TNBC, thereby promoting cell cycle progression and cell proliferation. This dual functionality highlights the complexity of PHF8 as an epigenetic regulator. Currently, the interplay between PHF8 and oncogenic transcription factors remains an area of active investigation. Because c-Myc is highly expressed in TNBC and is well-known for its role in the regulation of p27^kip1^ expression [65–71], we propose that PHF8 may cooperate with c-Myc to repress p27^kip1^ expression. We are investigating whether PHF8 directly interacts with c-Myc or other transcriptional regulators to modulate p27^kip1^ expression in TNBC. We are also exploring PHF8’s broader impact on transcriptional regulation in human cancers. In addition to its histone demethylase activity, PHF8 has been reported to target non-histone proteins, including transcription factors and chromatin remodelers. PHF8 interacts with DNA topoisomerase 2-binding protein 1 (TopBP1), leading to the suppression of DNA damage repair initiation. The dissociation of PHF8 from TopBP1 presents a potential strategy for developing novel cancer therapies targeting TopBP1 [72]. Thus, elucidating the underlying mechanisms of histone-dependent and -independent may reveal novel therapeutic targets and strategies aimed at disrupting PHF8-mediated oncogenic signaling pathways in TNBC.

During our investigations of the influence of HER3 signaling on PHF8, we noticed that the changes in PHF8 protein levels were more pronounced than that at the mRNA level. This discrepancy is likely because PHF8 expression is regulated through both post-transcriptional and post-translational mechanisms. Numerous studies have shown the non-transcriptional regulation of PHF8 [54, 73, 74]. For instance, miR-22 directly targets and suppresses PHF8 expression [55, 75]. Our studies discovered several miRNAs, including miR-22, Let-7-5p, and miR-34b-5p downregulated by HER3 signaling in TNBC cells. Further studies identified miR-34b-5p as a novel miRNA inhibiting PHF8 expression in TNBC. It has been shown that miR-34b-5p acts as a key modulator of multiple oncogenic pathways, particularly in cancers with compromised p53 activity [76, 77]. Since TNBC is frequently characterized by p53 mutations and dysregulated cell cycle control, we believe miR-34b-5p plays a critical role in this context. The precise mechanism by which HER3 signaling suppresses miR-34b-5p requires further investigation. Current literature suggests three potential regulatory pathways: (1) transcriptional repression through PI3K/AKT-mediated FOXO3a or p53 inhibition [78, 79], (2) post-transcriptional modulation via lncRNA sponging effects (e.g., LINC02418) [80], or (3) epigenetic silence involving DNA methyltransferase recruitment [81]. While our current data in TNBC models most strongly supports the transcriptional regulation hypothesis, particularly through the HER3/PI3K/AKT/FOXO3a axis, though other mechanisms may contribute. Further studies are needed to fully elucidate the underlying molecular pathways specific to the TNBC context. The miR-34 family, including miR-34b-5p, has been extensively studied for its tumor-suppressive properties. Restoring miR-34b-5p expression through miRNA replacement therapy may present a compelling strategy for TNBC treatment. Notably, MRX34, a miR-34a mimic, was the first tumor-targeted microRNA drug to enter a phase I clinical trial (NCT01829971), highlighting the therapeutic potential of miR-34 family members. Unfortunately, this clinical trial was terminated due to systemic toxicity towards the immune system [82]. Nonetheless, more detailed analysis is required to evaluate the feasibility and safety of miRNA-based therapies for cancer treatment.

## Conclusion

Our studies establish a novel HER3/miR-34b-5p/PHF8 signaling axis that drives TNBC progression. We demonstrate that elevated expression of HER3 or activation of HER3 signaling suppresses miR-34b-5p expression to upregulate PHF8 in TNBC cells. Increased PHF8 plays an essential role in HER3-driven TNBC cell proliferation and cell cycle progression via downregulation of p27^kip1^, a key inhibitor of CDKs in G1-S transition. Moreover, our study reveals a significantly positive correlation between HER3 and PHF8 in TNBC specimens and clinic relevance of the HER3/miR-34b-5p/PHF8 axis in association with the survival outcomes of breast cancer patients. Taken together, our findings not only improve our understanding of the molecular basis of HER3-driven TNBC progression, but they also support the notion that targeting HER3 with a blocking Ab or ADC, inhibition of PHF8, and/or leveraging miRNA replacement therapy may represent promising therapeutic strategies for patients with HER3-driven TNBC.

## Supplementary information

Supplementary data-

Original Data

## Data Availability

Data generated from the online tool GEPIA2, the Breast Cancer Gene-Expression Miner v5.2, TargetScan and Kaplan-Meier plotter databases are publicly available (http://gepia2.cancer-pku.cn/#index, https://kmplot.com/analysis/, http://bcgenex.ico.unicancer.fr/BC-GEM/GEM-Accueil.php?js=1, https://www.targetscan.org/vert_80/). The datasets generated and/or analyzed during this study are available from the corresponding author.

## References

[1] Dent R, Trudeau M, Pritchard KI, Hanna WM, Kahn HK, Sawka CA, et al. Triple-negative breast cancer: Clinical features and patterns of recurrence. Clin Cancer Res. 2007;13:4429–34. 17671126 10.1158/1078-0432.CCR-06-3045

[2] Araujo JM, De la Cruz-Ku G, Cornejo M, Doimi F, Dyer R, Gomez HL, et al. Prognostic capability of TNBC 3-gene score among triple-negative breast cancer subtypes. Cancers. 2022;14:4286. 36077821 10.3390/cancers14174286 PMC9454544

[3] Cai SL, Liu JJ, Liu YX, Yu SH, Liu X, Lin XQ, et al. Characteristics of recurrence, predictors for relapse and prognosis of rapid relapse triple-negative breast cancer. Front Oncol. 2023;13:1119611. 10.3389/fonc.2023.1119611 PMC9978400 36874102

[4] Ma D., Yang Q, Yin K, Shi P, Chen X, Dong T., et al. Analysis of the clinicopathological characteristics and prognosis of triple-positive breast cancer and HER2-positive breast cancer-a retrospective study. Front Oncol. 2023;12:999894. 10.3389/fonc.2022.999894 PMC9885258 36727058

[5] Gluz O, Nitz U, Kolberg-Liedtke C, Prat A, Christgen M, Kuemmel S, et al. De-escalated neoadjuvant chemotherapy in early triple-negative breast cancer (TNBC): impact of molecular markers and final survival analysis of the WSG-ADAPT-TN Trial. Clin Cancer Res. 2022;28:4995–5003. 35797219 10.1158/1078-0432.CCR-22-0482

[6] Lee J. Current treatment landscape for early triple-negative breast cancer (TNBC). J Clin Med. 2023;12:1524. 36836059 10.3390/jcm12041524 PMC9962369

[7] Foldi J, Geyer CE Jr. Precision medicine for metastatic TNBC: the FUTURE is now. Cell Res. 2023;33:491–2. 37156878 10.1038/s41422-023-00815-1 PMC10313756

[8] So JY, Ohm J, Lipkowitz S, Yang L. Triple negative breast cancer (TNBC): Non-genetic tumor heterogeneity and immune microenvironment: emerging treatment options. Pharm Ther. 2022;237:108253. 10.1016/j.pharmthera.2022.108253 PMC9378710 35872332

[9] Cerbone L, Blondeaux E, Boni L, Ruelle T, Russo S, Bonotto M, et al. Survival outcomes of triple-negative breast cancer (TNBC) patients in the pre-immunotherapy age: an analysis of Gruppo Italiano Mammella (GIM) 14 BIOMETA study with a focus on biological subtypes. Ann Oncol. 2021;32:S475–S476.

[10] Brockwell NK, Owen KL, Zanker D, Spurling A, Rautela J, Duivenvoorden HM, et al. Neoadjuvant interferons: critical for effective PD-1-based immunotherapy in TNBC. Cancer Immunol Res. 2017;5:871–84. 28848054 10.1158/2326-6066.CIR-17-0150

[11] Yang Y., Li H., Yang W, Shi Y. Improving efficacy of TNBC immunotherapy: based on analysis and subtyping of immune microenvironment. Front Immunol. 2024;15. 10.3389/fimmu.2024.1441667 PMC11487198 39430759

[12] Li Y, Miao W, Yuan C, Tang J, Zhong N, Jin Y, et al. PARP inhibitor boost the efficacy of photothermal therapy to TNBC through enhanced DNA damage and inhibited homologous recombination repair. Drug Deliv Transl Re. 2025;15:955–67. 10.1007/s13346-024-01650-6 38954244

[13] Dey N, Hui W, Sun Y, Elvin P, De P, Leyland-Jones B. Combination of PARP inhibitor, Olaparib with pathway -targeted drug, vandetanib (Caprelsa™) in TNBC: A proof of principle study to determine the role of PI3K-AKT-mTOR or RAS-MAPK pathways in targeted drug-response. Cancer Res. 2012;72:2250.

[14] Bardia A, Coates JT, Spring L, Sun S, Juric D, Thimmiah N, et al. Sacituzumab Govitecan, combination with PARP inhibitor, Talazoparib, in metastatic triplenegative breast cancer (TNBC): translational investigation. Cancer Res. 2022;82:2638.

[15] Lyu H, Han A, Polsdofer E, Liu S, Liu B. Understanding the biology of HER3 receptor as a therapeutic target in human cancer. Acta Pharm Sin B. 2018;8:503–10. 30109175 10.1016/j.apsb.2018.05.010 PMC6090011

[16] Ogden A, Bhattarai S, Sahoo B, Mongan NP, Alsaleem M, Green AR, et al. Combined HER3-EGFR score in triple-negative breast cancer provides prognostic and predictive significance superior to individual biomarkers. Sci Rep. 2020;10:3009. 32080212 10.1038/s41598-020-59514-1 PMC7033213

[17] Singer CF, Jahn SW, Hlauschek D, Heber UM, Mang-Manger C, Egle D, et al. HER2 and HER3 expression during neoadjuvant treatment of HER2-negative early breast cancer: potential for biomarker-driven sequencing of T-DXd and HER3-DXd. Cancer Commun. 2025;45:428–32. 10.1002/cac2.12657 PMC11999886 39757921

[18] Tanaka S, Suzuki E, Kotake T, Kawaoka S, Kawaguchi K, Nishimura T, et al. HER3 expression is associated with cold tumor immune microenvironment and the regulation of its signal in humanized breast cancer patient derived xenograft models converts to hot tumor. Cancer Res. 2022;82:6161.

[19] Mishra R, Kilroy MK, Patel H, Alanazi S, Garrett JT. Role of her3 mutations on breast cancer oncogenesis. Cancer Res. 2022;82:5412.

[20] Zhu M, Yu M, Meng Y, Yang J, Wang X, Li L, et al. HER3 receptor and its role in the therapeutic management of metastatic breast cancer. J Transl Med. 2024;22:665. 39020378 10.1186/s12967-024-05445-8 PMC11253420

[21] Tomasich E, Steindl A, Paiato C, Hatziioannou T, Kleinberger M, Berchtold L, et al. Frequent Overexpression of HER3 in Brain Metastases from Breast and Lung Cancer. Clin Cancer Res. 2023;29:3225–36. 37036472 10.1158/1078-0432.CCR-23-0020

[22] Yang L, Li Y, Shen E, Cao F, Li L, Li X, et al. NRG1-dependent activation of HER3 induces primary resistance to trastuzumab in HER2-overexpressing breast cancer cells. Int J Oncol. 2017;51:1553–62. 29048656 10.3892/ijo.2017.4130

[23] Rosato RR, Choi DS, Qian W, Chen W, Lantto J, Horak ID, et al. Pan-HER, an antibody mixture simultaneously targeting EGFR, HER2, and HER3, is highly effective in triple negative breast cancer patient-derived xenografts. Cancer Res. 2019;79:357.

[24] Lyu H, Hou D, Liu H, Ruan S, Tan C, Wu J, et al. HER3 targeting augments the efficacy of panobinostat in claudin-low triple-negative breast cancer cells. Npj Precis Oncol. 2023;7:72. 37537339 10.1038/s41698-023-00422-8 PMC10400567

[25] Lyu H, Shen F, Ruan S, Tan C, Zhou J, Thor AD, et al. HER3 functions as an effective therapeutic target in triple negative breast cancer to potentiate the antitumor activity of gefitinib and paclitaxel. Cancer Cell Int. 2023;23:204. 37716943 10.1186/s12935-023-03055-w PMC10504712

[26] Björkman M, Östling P, Härmä V, Virtanen J, Mpindi JP, Rantala J, et al. Systematic knockdown of epigenetic enzymes identifies a novel histone demethylase PHF8 overexpressed in prostate cancer with an impact on cell proliferation, migration and invasion. Oncogene. 2012;31:3444–56. 22120715 10.1038/onc.2011.512

[27] Kim JE, Pan X, Tse KY, Chan HH, Dong C, Huen MSY. PHF8 facilitates transcription recovery following DNA double-strand break repair. Nucleic Acids Res. 2024;52:10297–310. 39087553 10.1093/nar/gkae661 PMC11417394

[28] Qi HH, Sarkissian M, Hu GQ, Wang Z, Bhattacharjee A, Gordon DB, et al. Histone H4K20/H3K9 demethylase PHF8 regulates zebrafish brain and craniofacial development. Nature. 2010;466:503–7. 20622853 10.1038/nature09261 PMC3072215

[29] Liu S, Polsdofer EV, Zhou L, Ruan S, Lyu H, Hou D, et al. Upregulation of endogenous TRAIL-elicited apoptosis is essential for metformin-mediated antitumor activity against TNBC and NSCLC. Mol Ther Oncol. 2021;21:303–14. 10.1016/j.omto.2021.04.012 PMC8167201 34141868

[30] Lyu H, Huang J, He Z, Liu B. Targeting of HER3 with functional cooperative miRNAs enhances therapeutic activity in HER2-overexpressing breast cancer cells. Biol Proced Online. 2018;20:16. 30093840 10.1186/s12575-018-0081-x PMC6081814

[31] Lyu H, Wang S, Huang J, Wang B, He Z, Liu B. targeting miR-542-3p overcomes HER3 signaling-induced chemoresistance and enhances the antitumor activity of paclitaxel against HER2-overexpressing breast cancer. Cancer Lett. 2018;420:97–108. 29409974 10.1016/j.canlet.2018.01.065 PMC6089084

[32] Luo Z, Zhao Y, Azencott R. Impact of miRNA sequence on miRNA expression and correlation between miRNA expression and cell cycle regulation in breast cancer cells. Plos One. 2014;9:e95205. 24748078 10.1371/journal.pone.0095205 PMC3991594

[33] Kurozumi S, Yamaguchi Y, Kurosumi M, Ohira M, Matsumoto H, Horiguchi J. Recent trends in microRNA research into breast cancer with particular focus on the associations between microRNAs and intrinsic subtypes. J Hum Genet. 2017;62:15–24. 27439682 10.1038/jhg.2016.89

[34] Mohammadi-Yeganeh S, Hosseini V, Paryan M. Wnt pathway targeting reduces triple-negative breast cancer aggressiveness through miRNA regulation in vitro and in vivo. J Cell Physiol. 2019;234:18317–28. 30945294 10.1002/jcp.28465

[35] Stieg DC, Wang Y, Liu LZ, Jiang BH. ROS and miRNA dysregulation in ovarian cancer development, angiogenesis and therapeutic resistance. Int J Mol Sci. 2022;23:6702. 35743145 10.3390/ijms23126702 PMC9223852

[36] Ouyang B, Bi M, Jadhao M, Bick G, Zhang X. miR-205 regulates tamoxifen resistance by targeting estrogen receptor coactivator MED1 in human breast cancer. Cancers. 2024;16:3992. 39682180 10.3390/cancers16233992 PMC11640040

[37] Dong L, Chen F, Fan Y, Long J. MiR-34b-5p inhibits cell proliferation, migration and invasion through targeting ARHGAP1 in breast cancer. Am J Transl Res. 2020;12:269–80. 32051752 PMC7013215

[38] Guo X, Zhou Q, Su D, Luo Y, Fu Z, Huang L, et al. Circular RNA circBFAR promotes the progression of pancreatic ductal adenocarcinoma via the miR-34b-5p/MET/Akt axis. Mol Cancer. 2020;19:83. 32375768 10.1186/s12943-020-01196-4 PMC7201986

[39] Zhao J, Lin H, Huang K, Li S. Cancer-associated fibroblasts-derived extracellular vesicles carrying lncRNA SNHG3 facilitate colorectal cancer cell proliferation via the miR-34b-5p/HuR/HOXC6 axis. Cell Death Discov. 2022;8:346. 35922404 10.1038/s41420-022-01116-z PMC9349187

[40] De Pauw I, Wouters A, Van den Bossche J, Deschoolmeester V, Baysal H, Pauwels P, et al. Dual targeting of epidermal growth factor receptor and HER3 by MEHD7945A as monotherapy or in combination with cisplatin partially overcomes cetuximab resistance in head and neck squamous cell carcinoma cell lines. Cancer Biother Radio. 2017;32:229–38. 28910149

[41] Lee YH, Lee HJ, Kim HC, Lee Y, Nam SK, Hupperetz C. Short hairpin RNA-expressing oncolytic adenovirus-mediated HER3 inhibition enhances tumor growth inhibition and apoptosis in breast cancer. Mol Ther. 2022;30:579–92. -. 34628052 10.1016/j.ymthe.2021.10.004 PMC8821960

[42] Tao JJ, Castel P, Radosevic-Robin N, Elkabets M, Auricchio N, Aceto N, et al. Antagonism of EGFR and HER3 enhances the response to inhibitors of the PI3K-Akt pathway in triple-negative breast cancer. Sci Signal. 2014;7:ra29. 10.1126/scisignal.2005125 PMC4283215 24667376

[43] Takeda T, Tsubaki M, Genno S, Tokunaga K, Tanaka R, Nishida S. HER3/Akt/mTOR pathway is a key therapeutic target for the reduction of triple-negative breast cancer metastasis via the inhibition of CXCR4 expression. Int J Mol Med. 2023;52:80. 37477145 10.3892/ijmm.2023.5283 PMC10555474

[44] Würth R, Donato E, Michel LL, Saini M, Becker L, Cheytan T, et al. Circulating tumor cell plasticity determines breast cancer therapy resistance via neuregulin 1-HER3 signaling. Nat Cancer. 2025;6:67–85. 39753722 10.1038/s43018-024-00882-2 PMC11779641

[45] Sinevici N, Ataeinia B, Zehnder V, Lin K., Grove L, Heidari P, et al. HER3 differentiates basal from claudin type triple negative breast cancer and contributes to drug and microenvironmental induced resistance. Front Oncol. 2020;10:554704. 10.3389/fonc.2020.554704 PMC7715030 33330026

[46] Ogden A, Green A, Aleskandarany MA, Rakha E, Ellis IO, Li XX, et al. High HER3-EGFR score predicts aggressive disease course in triple-negative breast cancer. Lab Invest. 2016;96:63a-a.

[47] Tsai MS, Shamon-Taylor LA, Mehmi I, Tang CK, Lupu R. Blockage of heregulin expression inhibits tumorigenicity and metastasis of breast cancer. Oncogene. 2003;22:761–8. 12569369 10.1038/sj.onc.1206130

[48] Shu L, Chen A, Li L, Yao L, He Y, Xu J, et al. NRG1 regulates Fra-1 transcription and metastasis of triple-negative breast cancer cells via the c-Myc ubiquitination as manipulated by ERK1/2-mediated Fbxw7 phosphorylation. Oncogene. 2022;41:907–19. 34992218 10.1038/s41388-021-02142-4

[49] Krop IE, Masuda N, Mukohara T, Takahashi S, Nakayama T, Inoue K, et al. Patritumab deruxtecan (HER3-DXd), a human epidermal growth factor receptor 3-directed antibody-drug conjugate, in patients with previously treated human epidermal growth factor receptor 3-expressing metastatic breast cancer: a multicenter, phase I/II trial. J Clin Oncol. 2023;41:5550–60. 37801674 10.1200/JCO.23.00882 PMC10730028

[50] Brasó-Maristany F, Ferrero-Cafiero JM, Falato C, Martínez-Sáez O, Cejalvo JM, Margelí M, et al. Patritumab deruxtecan in HER2-negative breast cancer: part B results of the window-of-opportunity SOLTI-1805 TOT-HER3 trial and biological determinants of early response. Nat Commun. 2024;15:5826. 38992028 10.1038/s41467-024-50056-y PMC11239918

[51] Papa F, Grinda T, Rassy E, Cheickh-Hussin R, Ribeiro J, Antonuzzo L, et al. Long road towards effective HER3 targeting in breast cancer. Cancer Treat Rev. 2024;129:102786. 38885540 10.1016/j.ctrv.2024.102786

[52] Liu Q, Borcherding NC, Shao P, Maina PK, Zhang W, Qi HH. Contribution of synergism between PHF8 and HER2 signalling to breast cancer development and drug resistance. Ebiomedicine. 2020;51:102612. 31923801 10.1016/j.ebiom.2019.102612 PMC7000350

[53] Tong D, Liu Q, Liu G, Yuan W, Wang L, Guo Y, et al. The HIF/PHF8/AR axis promotes prostate cancer progression. Oncogenesis. 2016;5:e283. 27991916 10.1038/oncsis.2016.74 PMC5177772

[54] Lv Y, Shi Y, Han Q, Dai G. Histone demethylase PHF8 accelerates the progression of colorectal cancer and can be regulated by miR-488. Mol Med Rep. 2017;16:4437–44. 28765946 10.3892/mmr.2017.7130 PMC5647003

[55] Shao P, Liu Q, Maina PK, Cui J, Bair TB, Li T, et al. Histone demethylase PHF8 promotes epithelial to mesenchymal transition and breast tumorigenesis. Nucleic Acids Res. 2017;45:1687–702. 27899639 10.1093/nar/gkw1093 PMC5389682

[56] Ma S, Zhang J, Guo Q, Cao C, Bao K, Liu L, et al. Disrupting PHF8-TOPBP1 connection elicits a breast tumor-specific vulnerability to chemotherapeutics. Cancer Lett. 2022;530:29–44. 35051531 10.1016/j.canlet.2022.01.010

[57] Shi Y. Histone lysine demethylases: emerging roles in development, physiology and disease. Nat Rev Genet. 2007;8:829–33. 17909537 10.1038/nrg2218

[58] Liu W, Tanasa B, Tyurina OV, Zhou TY, Gassmann R, Liu WT, et al. PHF8 mediates histone H4 lysine 20 demethylation events involved in cell cycle progression. Nature. 2010;466:508–12. 20622854 10.1038/nature09272 PMC3059551

[59] Horton JR, Upadhyay AK, Qi HH, Zhang X, Shi Y, Cheng X. Enzymatic and structural insights for substrate specificity of a family of jumonji histone lysine demethylases. Nat Struct Mol Biol. 2010;17:38–43. 20023638 10.1038/nsmb.1753 PMC2849977

[60] Feng W, Yonezawa M, Ye J, Jenuwein T, Grummt I. PHF8 activates transcription of rRNA genes through H3K4me3 binding and H3K9me1/2 demethylation. Nat Struct Mol Biol. 2010;17:445–50. 20208542 10.1038/nsmb.1778

[61] Jin W, Ma Q, Chen Z, Jia G, Lu X, Xie X. The histone demethylase PHF8 promotes prostate cancer cell growth by activating the oncomiR miR-125b. Oncotargets Ther. 2015;8:1979–88. 10.2147/OTT.S85443 PMC4539089 26309412

[62] Hu Y, Mu H, Yang Y. Histone demethylase PHF8 promotes cell growth and metastasis of non-small-cell lung cancer through activating Wnt/β-catenin signaling pathway. Histol Histopathol. 2021;36:869–77. 34100557 10.14670/HH-18-349

[63] Tseng LL, Cheng HH, Yeh TS, Huang SC, Syu YY, Chuu CP, et al. Targeting the histone demethylase PHF8-mediated PKCα-Src-PTEN axis in HER2-negative gastric cancer. P Natl Acad Sci USA. 2020;117:24859–66. 10.1073/pnas.1919766117 PMC7547212 32958674

[64] Wu XN, Li J., He Q, Li B., He Y., Pan X, et al. Targeting the PHF8/YY1 axis suppresses cancer cell growth through modulation of ROS. Proc Natl Acad Sci USA. 2024;121:e2219352120. 10.1073/pnas.2219352120 PMC10786316 38165927

[65] Maina PK, Shao P, Liu Q, Fazli L, Tyler S, Nasir M, et al. c-MYC drives histone demethylase PHF8 during neuroendocrine differentiation and in castration-resistant prostate cancer. Oncotarget. 2016;7:75585–602. 27689328 10.18632/oncotarget.12310 PMC5342763

[66] Vlach J, Hennecke S, Alevizopoulos K, Conti D, Amati B. Growth arrest by the cyclin-dependent kinase inhibitor p27(Kip1) is abrogated by c-Myc. Embo J. 1996;15:6595–604. 8978686 PMC452484

[67] Bush AB, Baksh SM, Michaeli J. Regulation of p27 expression by c-MYC. Blood. 1999;94:138b-b.

[68] Khan F, Ricks-Santi LJ, Zafar R, Kanaan Y, Naab T. Expression of p27 and c-Myc by immunohistochemistry in breast ductal cancers in African American women. Ann Diagn Pathol. 2018;34:170–4. 29715580 10.1016/j.anndiagpath.2018.03.013 PMC6008231

[69] Li JC, Wang YJ, Wei SS, Xu S, Dai SL, Zhang L, et al. NEK2 promotes ESCC malignant progression by inhibiting cellular senescence via the FOXM1/c-Myc/p27 signaling pathway. Mol Carcinogen. 2024. 10.1002/mc.23839 39503194

[70] Lakis S, Koletsa T, Kostopoulos I, Papadimitriou C, Skarlos D, Koutras A, et al. p53, c-Myc and EGFR protein interactions confer worse prognosis in patients with triple negative breast cancer (TNBC). Cancer Res. 2013;73:P6-05-27.

[71] Tanaka S, Wariishi N, Kawaguchi-Sakita N, Adachi S, Toi M, Kamikubo Y. p300/CBP-c-Myc-survivin axis plays an important role in cell cycle and apoptosis in triple negative breast cancer (TNBC). Cancer Sci. 2021;112:446 -.

[72] Yuan L, Liang X, He L. Insights into the dissociation process and binding pattern of the BRCT7/8-PHF8 complex. ACS Omega. 2024;9:20819–31. 38764655 10.1021/acsomega.3c09433 PMC11097150

[73] Wang Q, Ma S, Song N, Li X, Liu L, Yang S, et al. Stabilization of histone demethylase PHF8 by USP7 promotes breast carcinogenesis. J Clin Invest. 2016;126:2205–20. 27183383 10.1172/JCI85747 PMC4887182

[74] Feng R, Li Z, Ge G, Wang C, Jia Y, Ouyang J. NEDD4L represses prostate cancer cell proliferation via modulating PHF8 through the ubiquitin-proteasome pathway. Clin Transl Oncol. 2023;25:243–55. 36136271 10.1007/s12094-022-02933-5

[75] Cai MZ, Wen SY, Wang XJ, Liu Y, Liang H. MYC regulates PHF8, which promotes the progression of gastric cancer by suppressing miR-22-3p. Technol Cancer Res T. 2020;19. 10.1177/1533033820967472 PMC7607725 33111613

[76] Zhang L, Liao Y, Tang L. MicroRNA-34 family: a potential tumor suppressor and therapeutic candidate in cancer. J Exp Clin Canc Res. 2019;38:53. 10.1186/s13046-019-1059-5 PMC6360685 30717802

[77] Bai X, Zheng L, Xu Y, Liang Y, Li D. Role of microRNA-34b-5p in cancer and injury: how does it work?. Cancer Cell Int. 2022;22:381. 36457043 10.1186/s12935-022-02797-3 PMC9713203

[78] Liu X, Feng J, Tang L, Liao L, Xu Q, Zhu S. The regulation and function of miR-21-FOXO3a-miR-34b/c signaling in breast cancer. Int J Mol Sci. 2015;16:3148–62. 25647415 10.3390/ijms16023148 PMC4346885

[79] Yang S, Zhu H, Cheng Q. Correlative analysis of miR-34b and p53 with pathological characteristics of NSCLC. Oncol Lett. 2019;17:5558–64. 31186777 10.3892/ol.2019.10239 PMC6507358

[80] Tian J, Cui P, Li Y, Yao X, Wu X, Wang Z, et al. LINC02418 promotes colon cancer progression by suppressing apoptosis via interaction with miR-34b-5p/BCL2 axis. Cancer Cell Int. 2020;20:460. 32973404 10.1186/s12935-020-01530-2 PMC7507712

[81] Yang C, Lu W, He H, Liu H. Inflammation and DNA methylation-dependent down-regulation of miR-34b-5p mediates c-MYC expression and CRL4(DCAF4) E3 ligase activity in colitis-associated cancer. Am J Pathol. 2020;190:674–88. 31972160 10.1016/j.ajpath.2019.11.013

[82] Hong DS, Kang YK, Borad M, Sachdev J, Ejadi S, Lim HY, et al. Phase 1 study of MRX34, a liposomal miR-34a mimic, in patients with advanced solid tumours. Brit J Cancer. 2020;122:1630–7. 32238921 10.1038/s41416-020-0802-1 PMC7251107

