# Supplementary material for: HER3 promotes triple-negative breast cancer progression by upregulating PHF8 via miR-34b-5p-dependent mechanism
Source: Cell Death Dis. 2025 Nov 6;16(1):802. doi: 10.1038/s41419-025-08115-9 (PMC12592479; doi:10.1038/s41419-025-08115-9)
Supplement: Supplementary file 1 — Supplementary data- [file 41419_2025_8115_MOESM1_ESM.pdf]

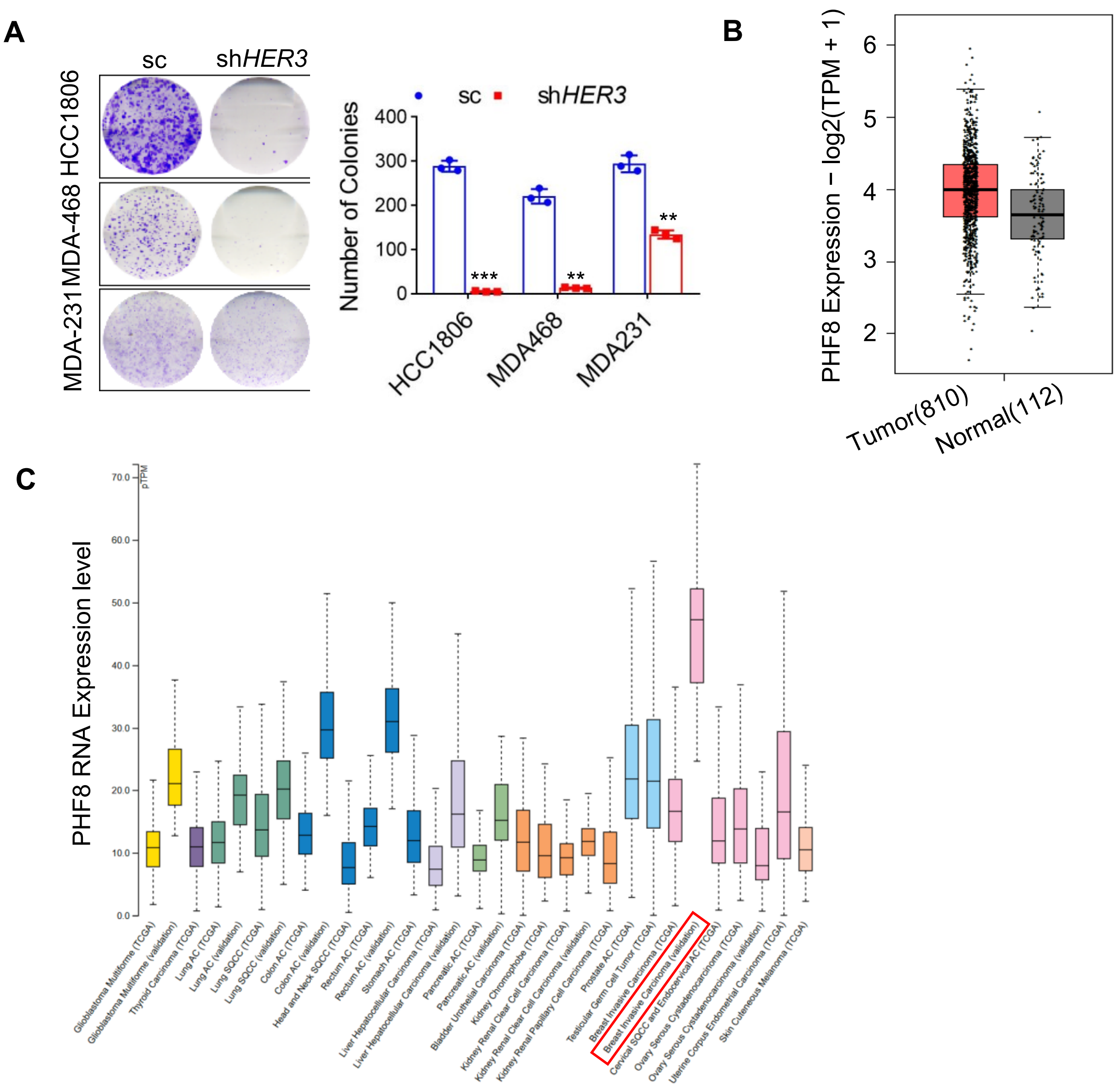

Fig S1: A, HCC1806, MDA-MB-468 and MDA-MB-231 cells infected with lentivirus containing either control shRNA (sc) or HER3-specific shRNA (sh*HER3*) were seeded into 6-well plates for colony formation assays. Representative images of cell colonies were captured and the colony numbers were quantified using ImageJ software. Error bars represent the standard deviation (SD) from three independent experiments. *, $p < 0.05$; **, $p < 0.01$; ***, $p<0.001$. **B&C,** Analysis of TCGA datasets showing PHF8 RNA expression levels in normal breast tissue versus breast cancer (B) and across various cancer types (C).

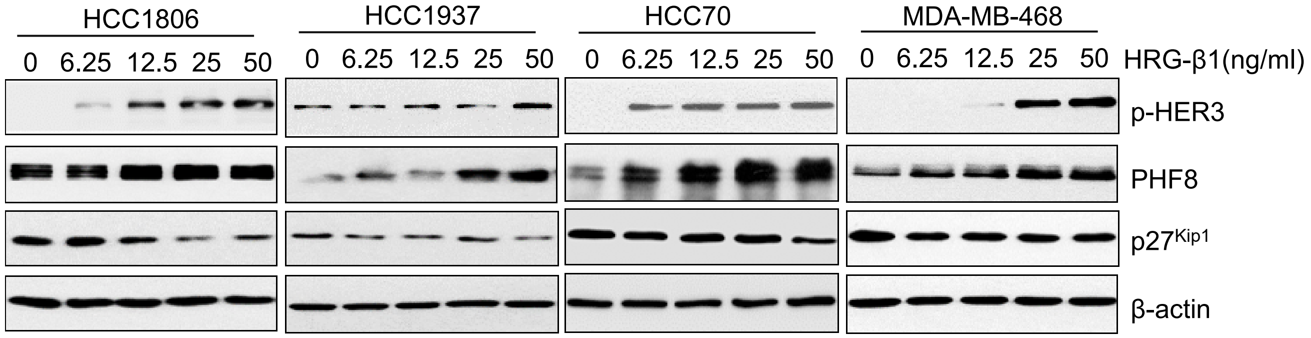

Fig S2: TNBC cells （HCC1806,HCC1937,HCC70 and MDA-MB-468） were seeded in 6-well plates and cultured overnight, followed by stimulation with HRG-β1 at the indicated concentrations for 24 hours. Cells were then harvested for Western blot analysis of p-HER3, PHF8, p27kip1, and β-actin.

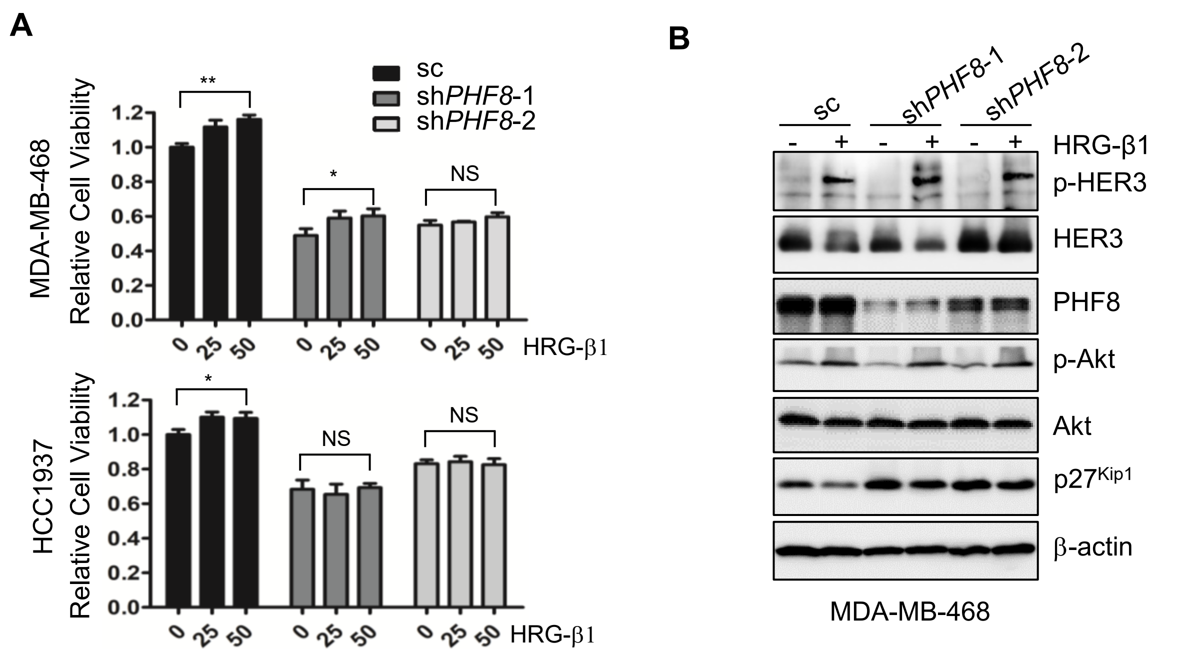

Fig S3: A, TNBC cells (MDA-MB-468, HCC1937) were plated in 96-well plates and incubated overnight at 37°C with 5% CO_2. The medium was then replaced with 0.1 mL fresh medium containing 0.5% FBS, with or without the indicated concentrations of HRG-β1, and incubated for an additional 72 hours. Cell viability was assessed using MTS assays, and results are shown as relative viability in bar graphs. **B,** MDA-MB-468 cells were seeded into 6-well plates and transduced with lentivirus containing either control shRNA (sc) or PHF8-specific shRNA (sh1 and sh2) for 24 hours. Subsequently, the cells were stimulated with or without HRG-β1 (25 ng/ml) for additional 24 hours. Cells were collected for western blot analysis of p-HER3, HER3, PHF8, p-Akt, Akt, p27kip1, and β-actin.

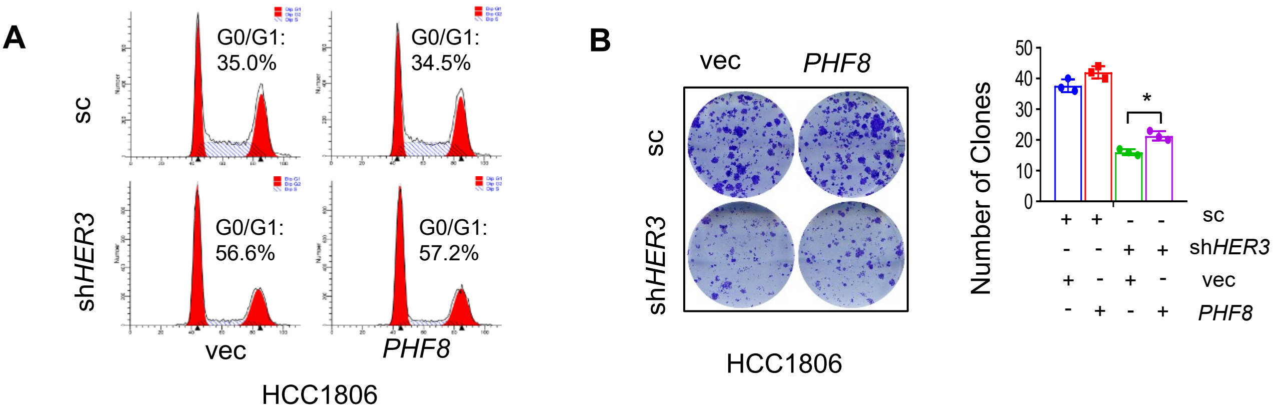

Fig S4: HCC1806 cells were infected with lentivirus containing either control shRNA (sc) or HER3-specific shRNA (shHER3) to achieve knockdown of HER3. The cells were then transfected with either a control vector (vec) or PHF8 cDNA expression vector (PHF8). **A,** The resulting cells were analyzed by flow cytometry to assess cell cycle distribution. **B,** and by colony formation assays. Representative images of the colonies were captured, and the colony numbers were quantified using ImageJ software

A

Co-Expression Table of Pan-Cancer Analysis for hsa-miR-34b-5p and PHF8

Show 10 entries Search:

Cancer	CancerFullName	SampleNum	Coefficient-R	p-value
LGG	Brain Lower Grade Glioma	525	-0.157	3.11e-4
PRAD	Prostate Adenocarcinoma	495	-0.158	4.28e-4
BLCA	Bladder Urothelial Carcinoma	408	-0.141	4.31e-3
LUAD	Lung Adenocarcinoma	512	-0.111	1.19e-2
OV	Ovarian Serous Cystadenocarcinoma	376	0.099	5.56e-2
LAML	Acute Myeloid Leukemia	83	-0.179	1.06e-1
UCEC	Uterine Corpus Endometrial Carcinoma	538	0.069	1.12e-1
STAD	Stomach Adenocarcinoma	372	-0.082	1.16e-1
PAAD	Pancreatic Adenocarcinoma	178	0.118	1.17e-1
THYM	Thymoma	119	0.118	2.02e-1

B

Co-Expression Table of Pan-Cancer Analysis for hsa-miR-34b-5p and HER3

Show 10 entries Search:

Cancer	CancerFullName	SampleNum	Coefficient-R	p-value
LGG	Brain Lower Grade Glioma	525	0.227	1.46e-7
ESCA	Esophageal Carcinoma	162	-0.375	9.03e-7
KIRC	Kidney Renal Clear Cell Carcinoma	517	-0.144	1.02e-3
BLCA	Bladder Urothelial Carcinoma	408	-0.135	6.19e-3
ACC	Adrenocortical Carcinoma	79	-0.291	9.27e-3
PRAD	Prostate Adenocarcinoma	495	-0.112	1.28e-2
UCS	Uterine Carcinosarcoma	56	0.317	1.72e-2
THCA	Thyroid Carcinoma	509	0.104	1.93e-2
BRCA	Breast Invasive Carcinoma	1085	0.062	3.98e-2
PCPG	Pheochromocytoma and Paraganglioma	183	0.148	4.50e-2

Fig S5: Co-Expression Analysis for hsa-miR-34b-5p and PHF8 (A), hsa-miR-34b-5p and HER3(B) using ENCORI/starBase (Encyclopedia of RNA Interactomes) online tool.

PHF8	HER3		
	High	Low	Total
High	24	17	41
Low	8	42	50
Total	32	59	91

Fig S6: Association between HER3 and PHF8 expression levels in 91 TNBC specimens. The table compares high/low HER3 expression (rows) with high/low PHF8 expression (columns), showing patient counts in each category. "High” expression was defined as ≥++.

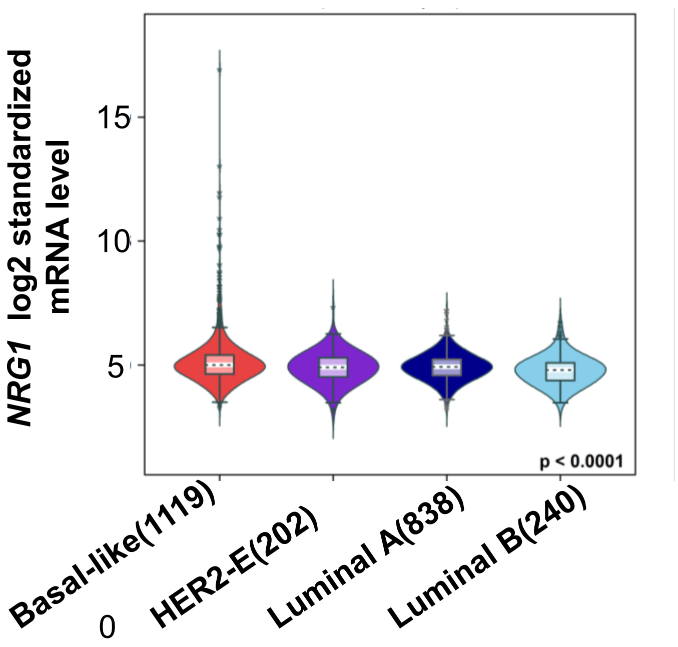

Dunnett-Tukey-Kramer's test:

gene-expression comparisons			p-value
Basal-like	>	HER2-E	< 0.0001
Basal-like	>	Luminal A	< 0.0001
Basal-like	>	Luminal B	< 0.0001

Fig S7: NRG1 mRNA expression levels across different breast cancer subtypes were analyzed using the Breast Cancer Gene-Expression Miner v5.2 online tool.
