# Supplementary material for: HER3 promotes triple-negative breast cancer progression by upregulating PHF8 via miR-34b-5p-dependent mechanism
Source: Cell Death Dis. 2025 Nov 6;16(1):802. doi: 10.1038/s41419-025-08115-9 (PMC12592479; doi:10.1038/s41419-025-08115-9)
Supplement: Supplementary file 2 — Original Data [file 41419_2025_8115_MOESM2_ESM.pdf]

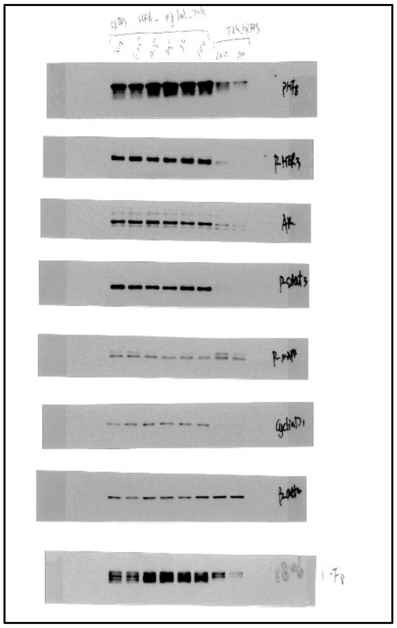

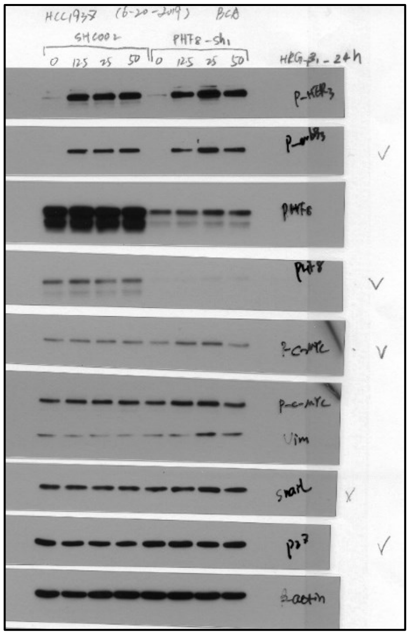
HCC1937 (6-20-2019) BCA
SHC002
PHF8-sh1
0 12.5 25 50 0 12.5 25 50
p-HER3
PHF8
p27
β-actin

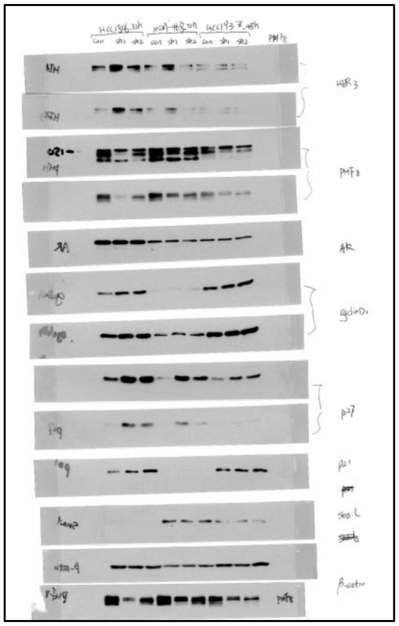

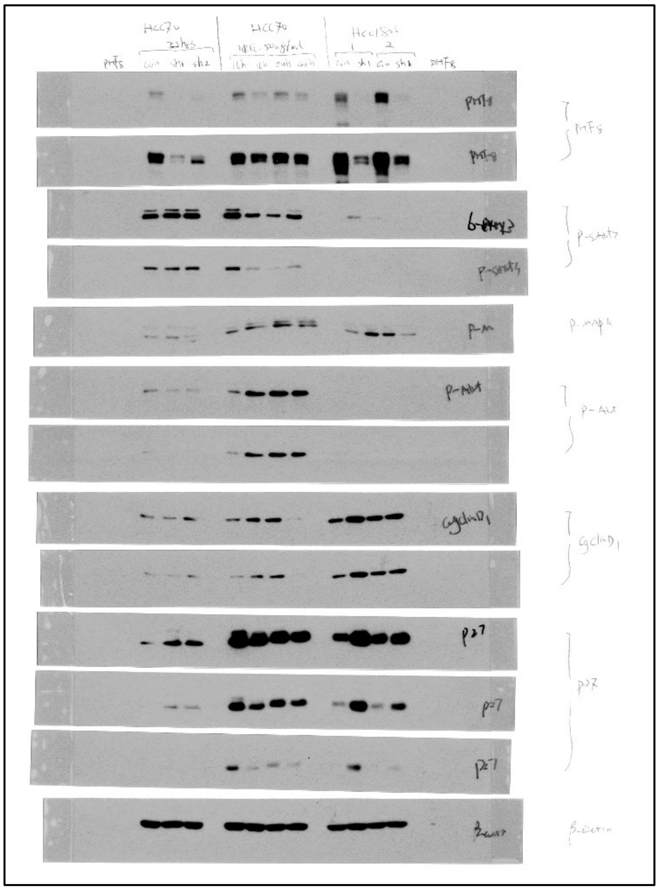

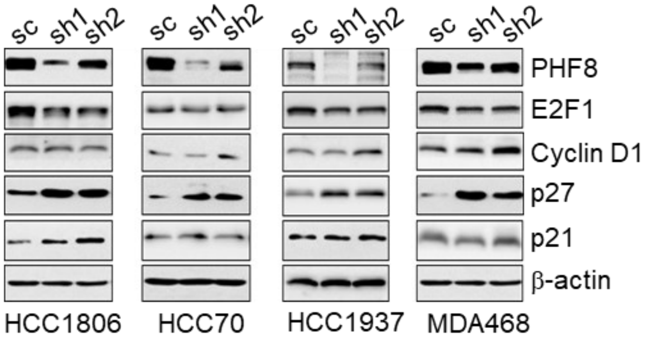
sc sh1 sh2
PHF8
E2F1
Cyclin D1
p27
p21
β-actin
HCC1806
HCC70
HCC1937
MDA468

Fig 2C

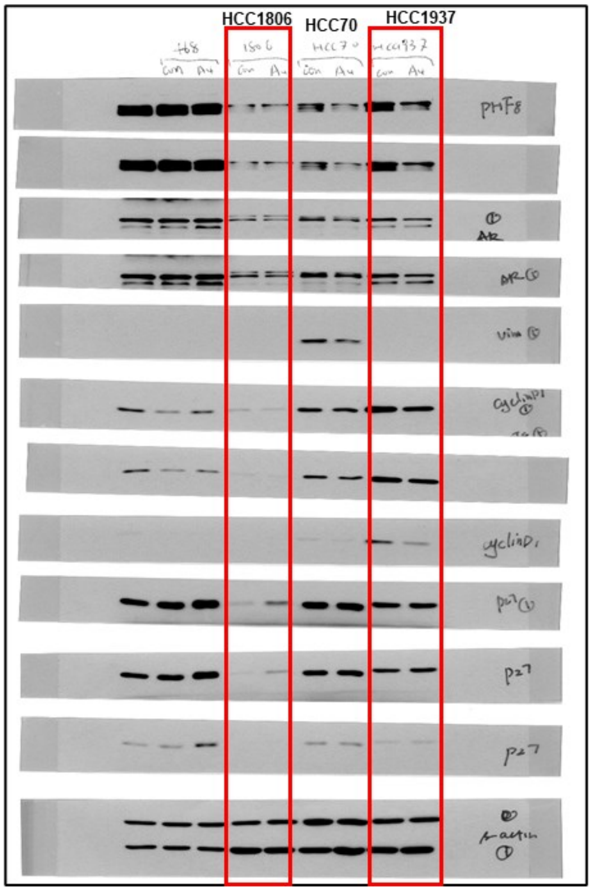

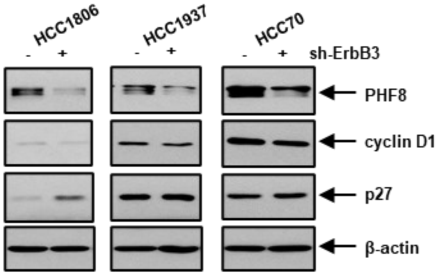

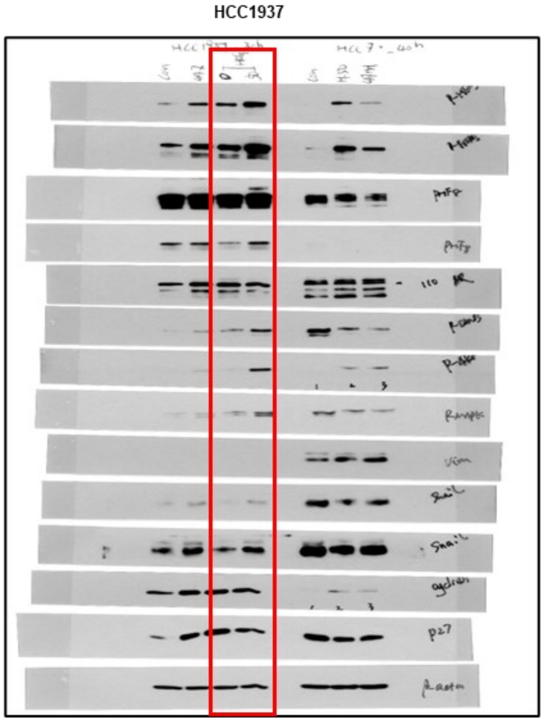

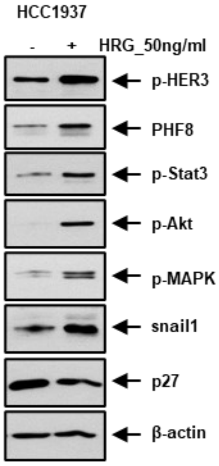

Knockdown HER3 (labeled as A4) or HRG stimulated HER3 signaling in TNBC cells

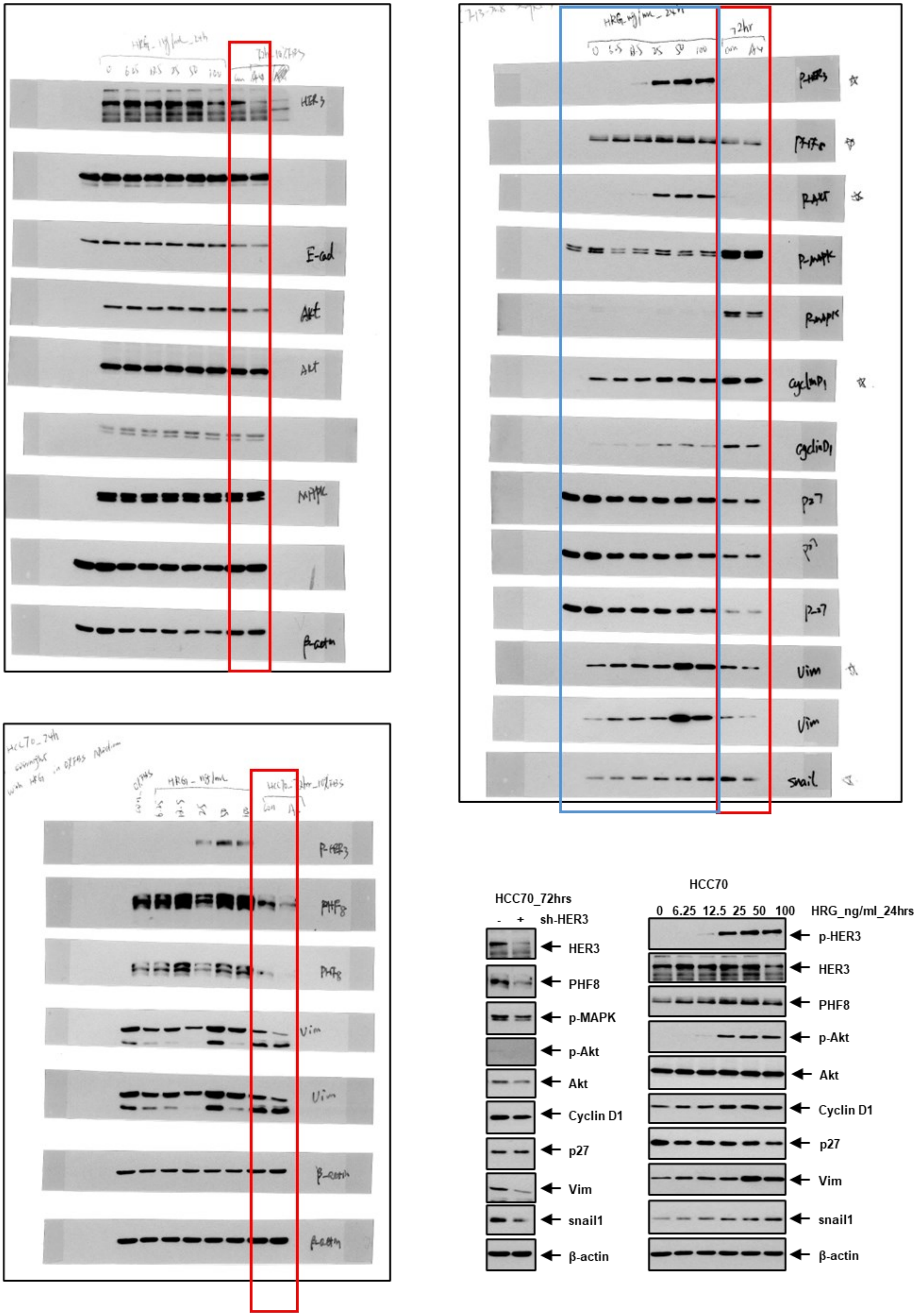

Knockdown HER3 (labeled as A4) or HRG stimulated HER3 signaling in TNBC cells

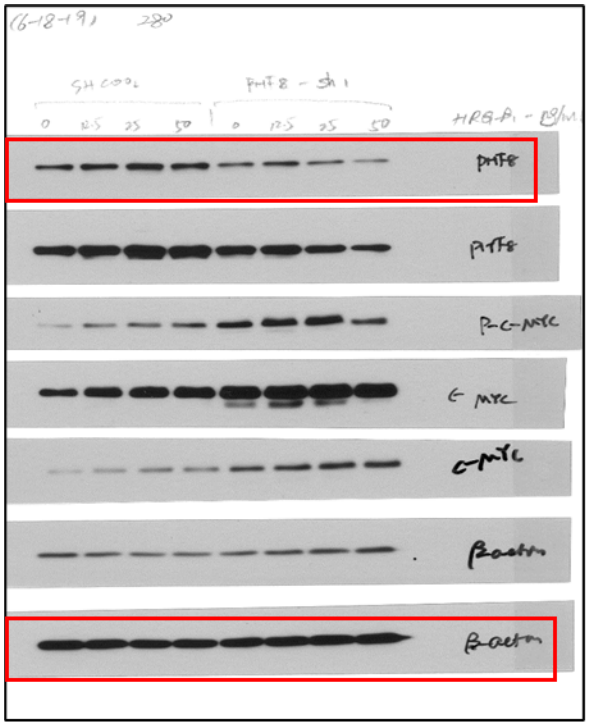

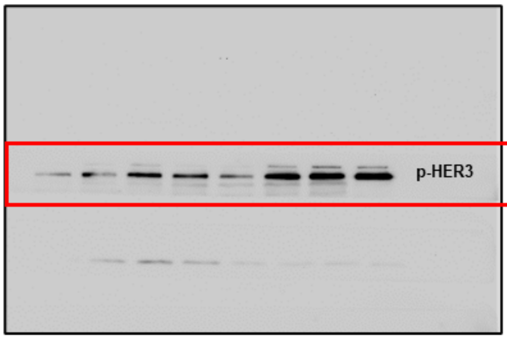

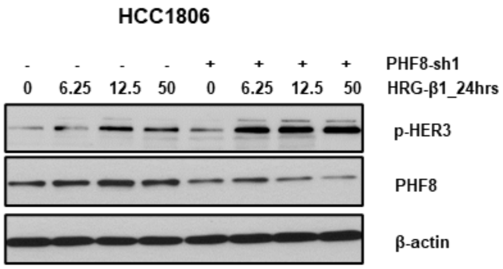

HRG stimulated HER3 signaling in TNBC cells

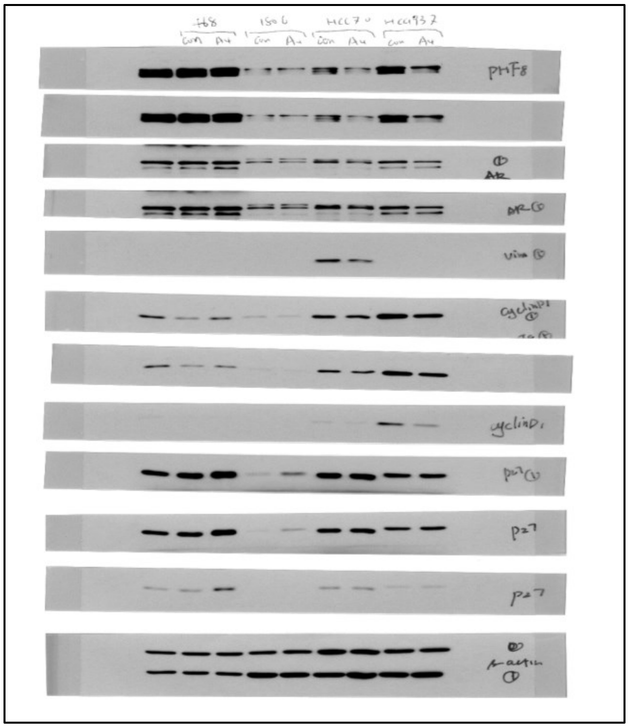

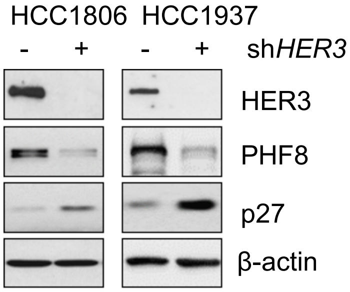

HCC1806 HCC1937
- + - + sh*HER3*
HER3
PHF8
p27
β-actin

Fig 2E

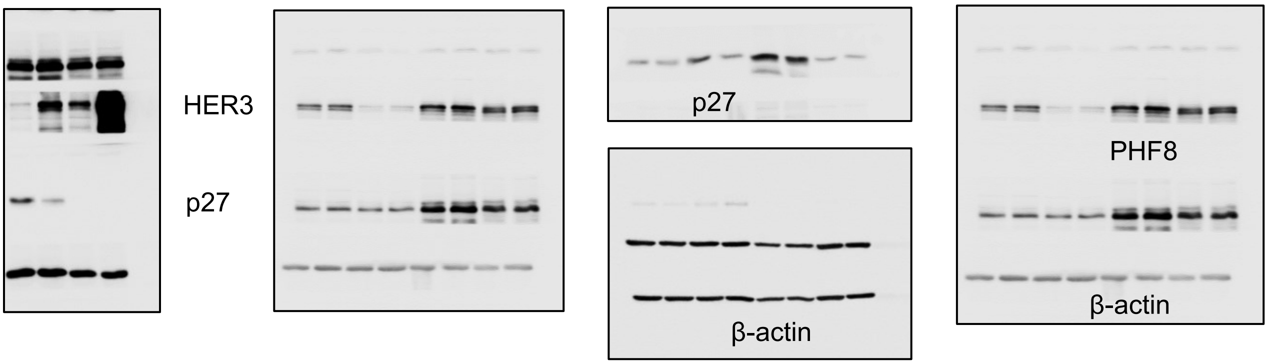

HER3
p27
p27
PHF8
β-actin
β-actin

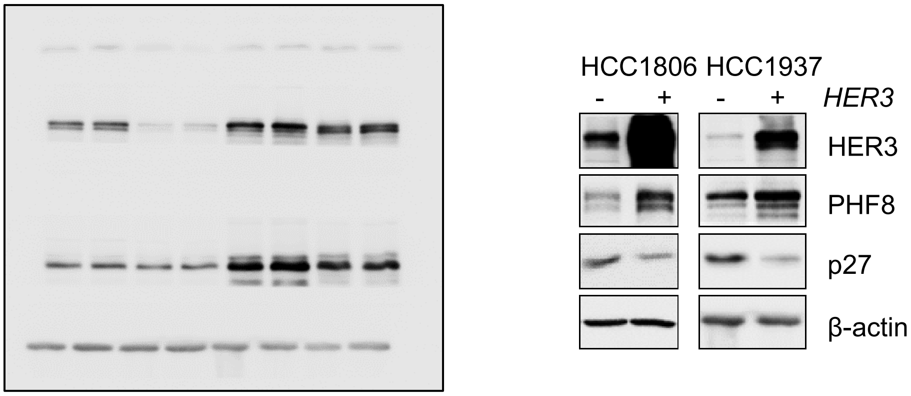

HCC1806 HCC1937
- + - + *HER3*
HER3
PHF8
p27
β-actin

Fig 2G

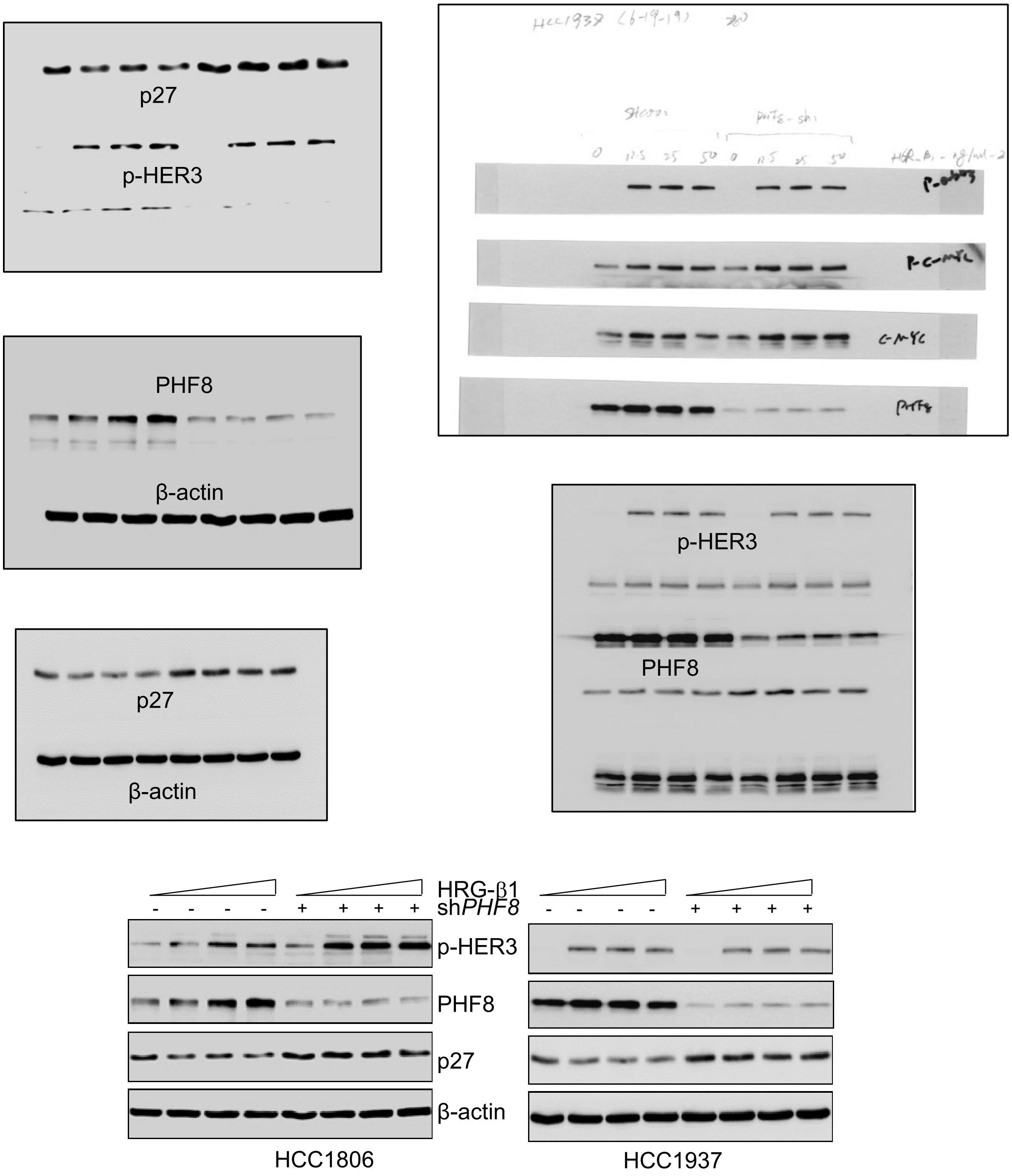
p27
p-HER3
PHF8
β-actin
p27
β-actin
p-HER3
PHF8
HRG-β1
shPHF8
- - - - + + + +
- - - - + + + +
p-HER3
PHF8
p27
β-actin
HCC1806
HCC1937

Fig 2H

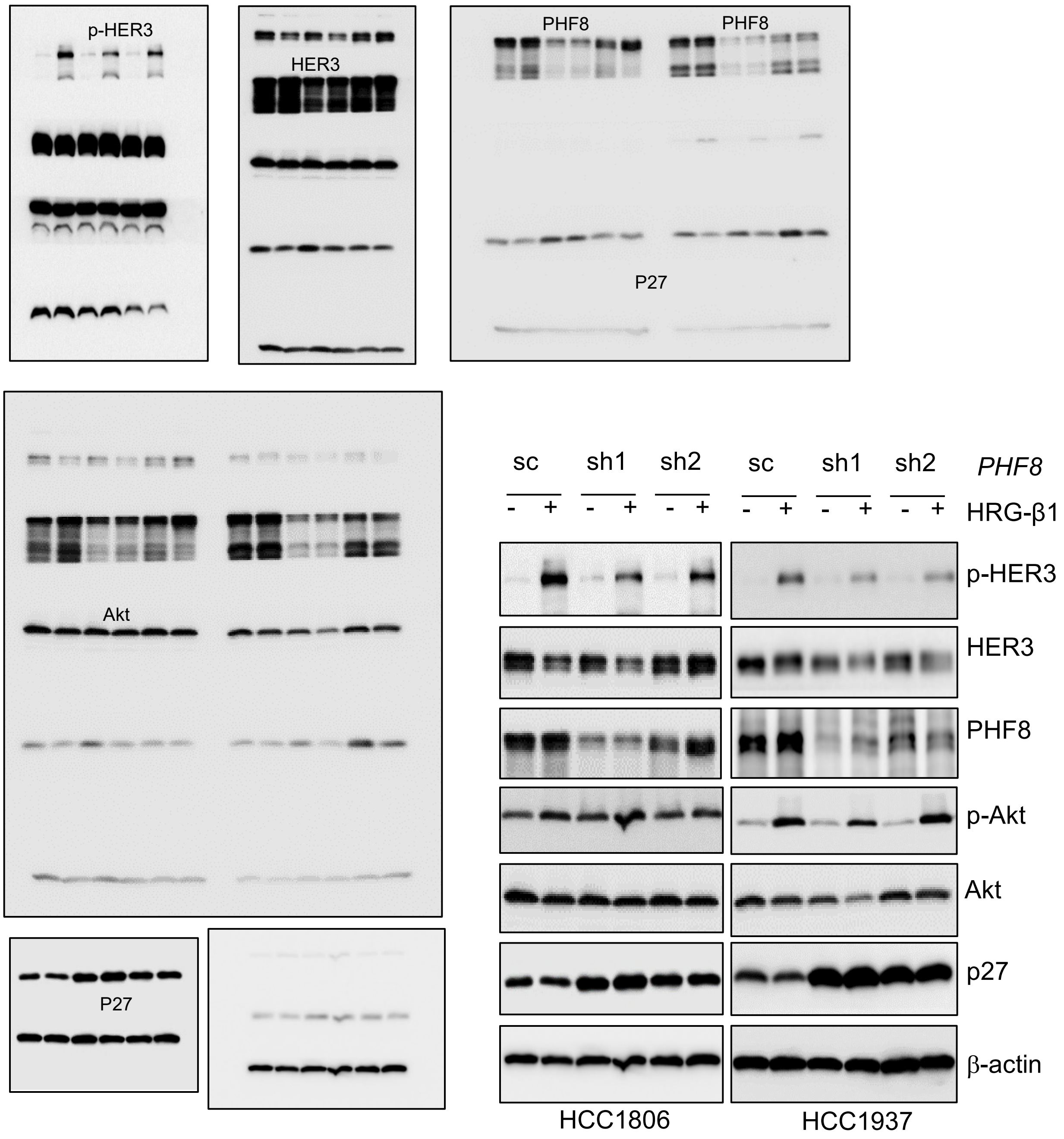
p-HER3
HER3
PHF8
PHF8
P27
Akt
P27
sc sh1 sh2 sc sh1 sh2 PHF8
- + - + - + - + - + - + HRG-β1
p-HER3
HER3
PHF8
p-Akt
Akt
p27
β-actin
HCC1806
HCC1937

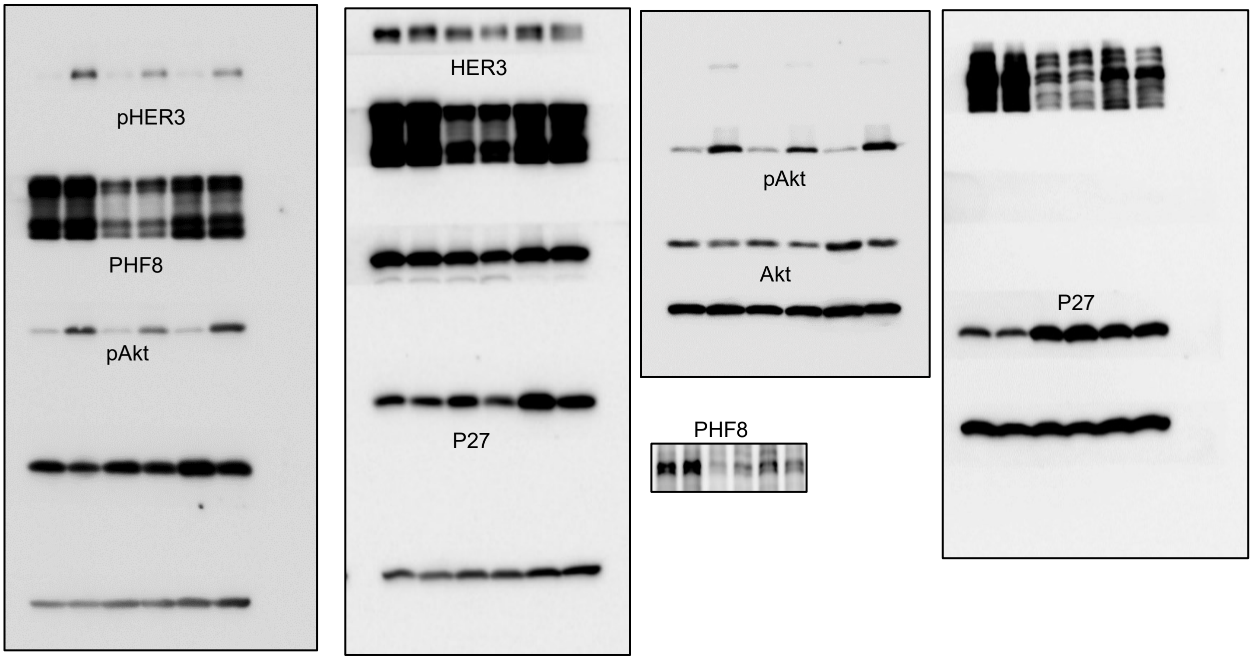

Fig 3C

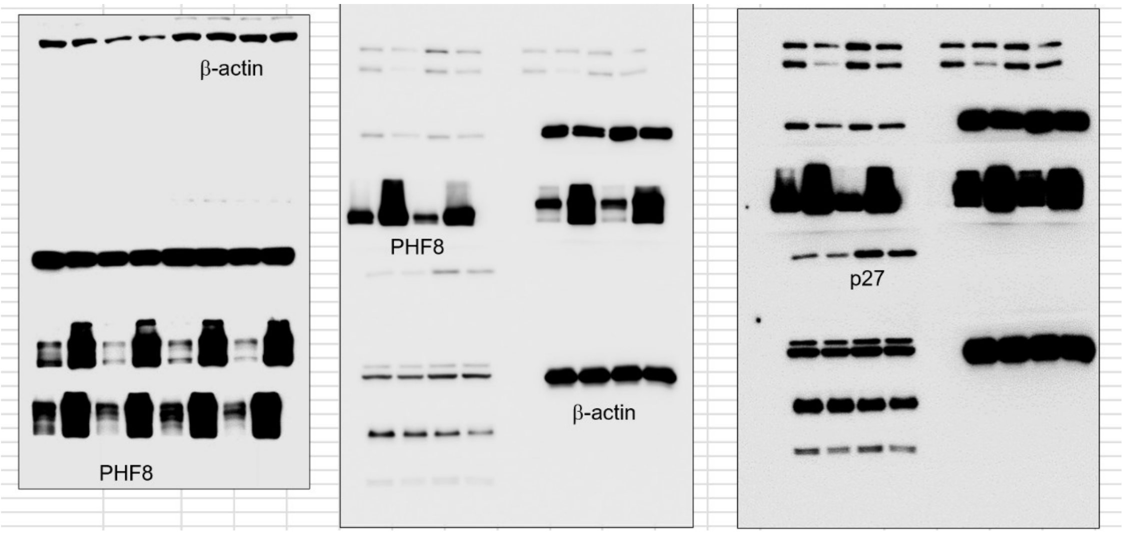
β-actin
PHF8
PHF8
β-actin
p27

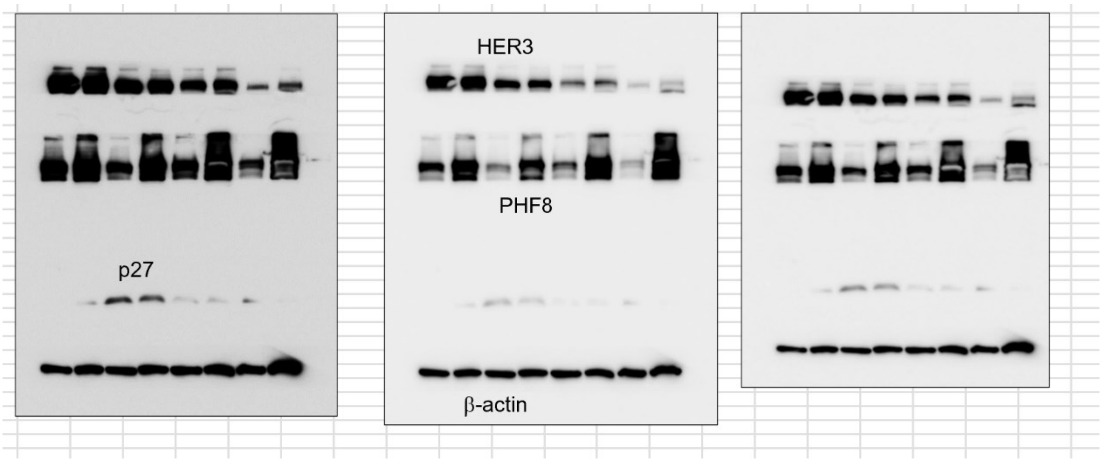
p27
HER3
PHF8
β-actin

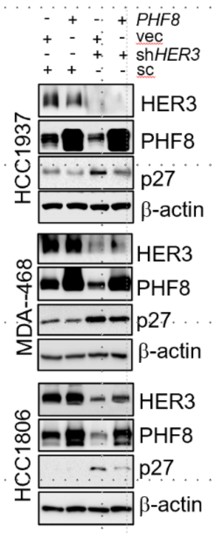
- + - + PHF8
+ - + - vec
- - + + shHER3
+ + - - sc
HCC1937
HER3
PHF8
p27
β-actin
MDA-468
HER3
PHF8
p27
β-actin
HCC1806
HER3
PHF8
p27
β-actin

Fig 3E

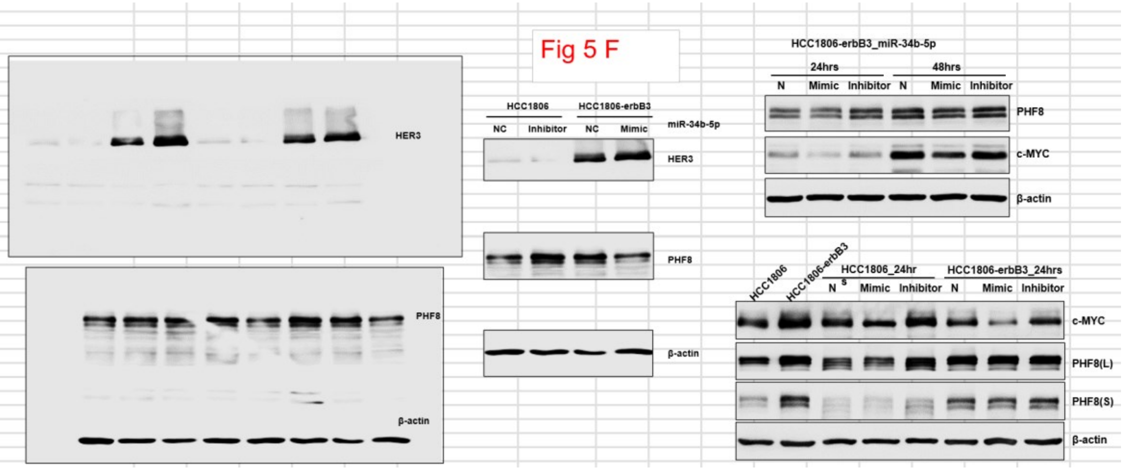
HER3
PHF8
β-actin
Fig 5 F
HCC1806
HCC1806-erbB3
NC
Inhibitor
NC
Mimic
miR-34b-5p
HER3
PHF8
β-actin
HCC1806-erbB3_miR-34b-5p
24hrs
48hrs
N
Mimic
Inhibitor
N
Mimic
Inhibitor
PHF8
c-MYC
β-actin
HCC1806
HCC1806-erbB3
HCC1806_24hr
HCC1806-erbB3_24hrs
N s
Mimic
Inhibitor
N
Mimic
Inhibitor
c-MYC
PHF8(L)
PHF8(S)
β-actin
